# Suppressing loop current of shielded loops at fundamental resonance

**DOI:** 10.1038/s41598-026-36956-7

**Published:** 2026-02-11

**Authors:** Wenjun Wang, Rasmus Alexander Jepsen, Juan Diego Sánchez-Heredia, Vitaliy Zhurbenko, Jan Henrik Ardenkjær-Larsen

**Affiliations:** 1 Technical University of Denmark, 2800 Kongens Lyngby, Denmark; 2 Technical University of Cartagena, 30202 Cartagena, Spain

**Keywords:** Decoupling, Loop antenna, Coil, Magnetic resonance imaging (MRI), Shielded loop, Biomedical engineering, Electrical and electronic engineering

## Abstract

In magnetic resonance imaging (MRI), arrays of small loop antennas/coils are extensively used for signal reception. In such arrays, the loop current must be suppressed to prevent transmit antennas or other elements in a receive array from being detuned, thereby preserving image quality. While suppressing the current in conventional loop antennas/coils is straightforward, achieving effective suppression in shielded loops has remained an unresolved challenge. In this article, we derive theoretical principles for optimally suppressing the loop current of shielded loops at fundamental resonance and verify them experimentally. Maximal current suppression is achieved by carefully selecting the electrical load at the antenna outputs. We identify a critical relationship between loop inductance and load inductance specific to shielded loops. Our results demonstrate that the optimal suppression method reduces loop current by an additional 31–36 dB compared with the straightforward yet suboptimal approach of shorting the antenna outputs. These findings can facilitate the development of more robust antenna/coil arrays for MRI applications.

## Introduction

A loop antenna consists of one or more turns of conductive wire wound around a frame of any shape, which is typically circular^1^. Loop antennas may be formed from single-conductor wires or two-conductor coaxial cables. While electrically small loop antennas are inefficient far-field radiators, they are valuable for measuring magnetic fields and current distributions. In MRI, electrically small loop antennas are widely used for signal reception and are commonly referred to as “coils”. In this article, loop antennas formed from single-conductor wires are termed “loop antennas” or “coils”, often written together as “loop antennas/coils”. Loop antennas formed from two-conductor coaxial cables are called “shielded loops”.

Loop antennas have existed since 1900^2^ and have been used in MRI since its inception in the 1970s^3^. Modern coaxial-cable shielded loops were invented in 1941^4^ and introduced into MRI in 2018^5^. Prior to their adoption in MRI, shielded loops had already been widely utilized for magnetic field sensing^6–8^ and could take various forms, including multi-turn multi-gap^9,10^ and Möbius loops^11^. Since their introduction into MRI, shielded loops have demonstrated greater resilience to mechanical deformation and exhibit lower noise correlation than loop antennas/coils when assembled into arrays^5,12–15^. When used in MRI, shielded loops are typically designed to operate at fundamental resonance, where their impedance characteristics closely resemble those of parallel R-L-C resonant circuits and exhibit high impedance at their terminals. The MRI community therefore refers to them as “high-impedance coils” or “parallel-resonant coils”.

The imaging process of MRI involves a transmission stage and a reception stage. To facilitate transmission and reception, at least one radio frequency (RF) transmitter and one RF receiver are needed, the latter of which is typically an electrically small loop antenna/coil or a loop antenna/coil array as described above. During transmission, the RF transmitter generates an alternating magnetic field that excites the nuclear spins within the sample. This causes the macroscopic magnetic moment to rotate away from its thermal equilibrium orientation. During reception, the transmitter is deactivated. The sample magnetization relaxes toward equilibrium while inducing a voltage across the RF receiving loops, and accordingly, currents on these loops^16,17^. For standalone loops, there are no constraints on the loop current, but for loops in an array, the loop current must be suppressed. There are three reasons. First, in a receiving loop array, the currents of the loop antennas/coils interfere with one another through their magnetic fields. This interference couples the electrical characteristics of each element and merges their individual sensitivity profiles, which in practice significantly complicates the tuning of receiving circuitry and degrades image quality^18^. Second, the loop current may be induced by the high-power RF transmitter during the transmission phase. This can interfere with the nuclear spins in the sample, resulting in artifacts and a reduced signal-to-noise ratio (SNR) in the images^19^. Third, the current may pose a safety hazard.

By carefully overlapping loops, the mutual inductance between them can be minimized. However, this approach does not mitigate interference between non-adjacent loops, nor does it suppress loop current during the transmit phase. For these reasons, loop current suppression must be addressed in MRI. Such techniques have been described extensively in the literature. One technique is “preamplifier decoupling”, which employs specially designed preamplifiers (low-noise amplifiers) and antenna/coil-to-amplifier matching circuitry to suppress loop current^18,20^, thereby reducing inter-element coupling in antenna/coil arrays. Another technique is “active detuning”, which incorporates current-blocking circuits that activate during the transmit phase. In both cases, the key principle is to create a high-impedance block along the loop current path.

For loop antennas/coils, the method to suppress current is straightforward: connect a high-impedance load to the antenna/coil terminals^18,21^. For shielded loops, however, the method remains unclear. Short-circuiting the antenna/coil output terminals initially seemed intuitive^5^. However, whether through “reverse preamplifier decoupling”^5^ or through more rigorous analyses^22,23^, inductors have emerged as central to shielded loop current suppression. To date, discussions have only encompassed shielded loops with a single gap and without tuning components. For multi-gap loops^9^ or loops with tuning components^14,24^, a solution has yet to be proposed.

To tackle the problem of optimal loop current suppression, we propose suitable conditions for suppressing the loop current of a shielded loop at fundamental resonance. The main propositions are:

**Fig. 1:**
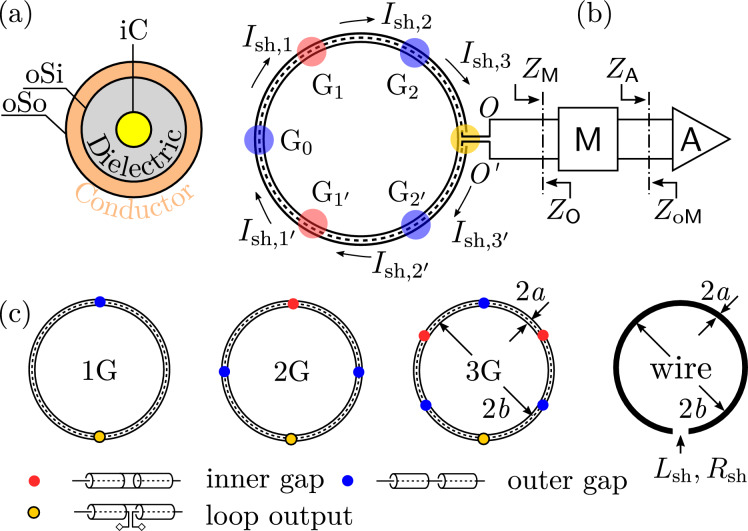
(**a**) Dissection of a shielded loop: iC—inner conductor, oSi—inner surface of the outer shield, oSo—outer surface of the outer shield. (**b**) Shielded loop terminated by a matching network $\mathsf{M}$ and an amplifier $\mathsf{A}$. The nodes of the antenna output are labeled $O$, $O^\prime$. Gaps are labeled $\textrm{G}_0, \textrm{G}_1, \textrm{G}_{1^\prime }$, etc. The current branches flowing on oSo are labeled $I_{\textrm{sh},1},I_{\textrm{sh},1^\prime }$, etc. (**c**) One-turn 1-gap, 2-gap, and 3-gap shielded loops, whose the loop diameter is $2a$ and the wire diameter is $2b$. A wire loop of loop diameter $2a$ and wire diameter $2b$ is drawn for comparison. The inductance and resistive loss of such a wire loop are denoted as ${L}_\textrm{sh}$ and ${R}_\textrm{sh}$ respectively. Subfigure (c) is re-used from Wang et al.^35^.

**Fig. 2:**
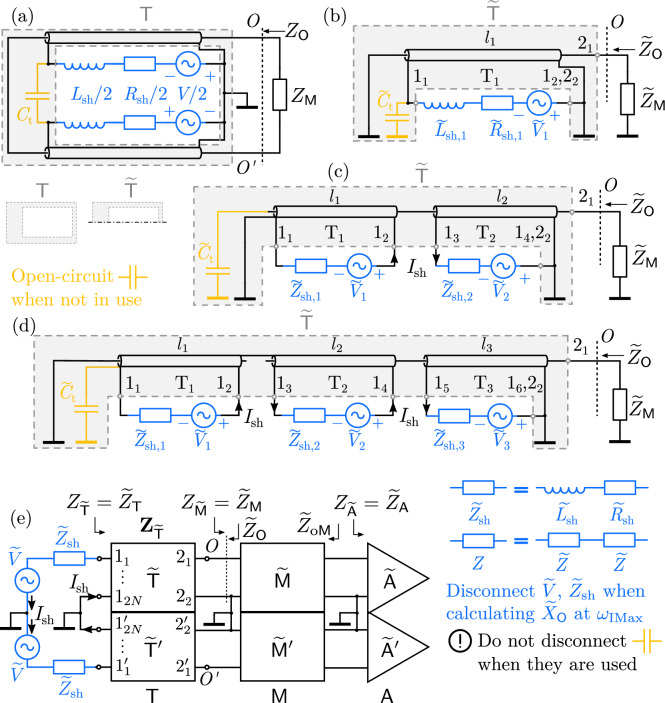
(**a**) Equivalent circuit of a 1-gap shielded loop. $C_\textrm{t}$ is an optional tuning component that lowers the resonant frequency $\omega _\textrm{IMax}$. $Z_\mathsf{M}$ is the Thévenin impedance of $\mathsf{M}$. $OO^\prime$ is the antenna output. The transmission lines and $C_\textrm{t}$ constitute a network $\mathsf{T}$, of which the half is denoted as $\widetilde{\mathsf{T}}$. (**b**) Half of the equivalent circuit of a 1-gap shielded loop. (**c**) Half of the equivalent circuit of a 2-gap shielded loop. (d) Half of the equivalent circuit of a 3-gap shielded loop. In (b), (c), and (d), transmission line $\textrm{T}_k$ has length $l_k$. All $l_k$’s need not be equal. Transmission lines $\textrm{T}_1,\dots ,\textrm{T}_N$ and $\widetilde{C}_\textrm{t}$ constitute a network $\widetilde{\mathsf{T}}$. Because all the currents flowing into and out of the network are equal to ${I}_\textrm{sh}$, the network can be deemed as having two ports the nodes of port 1 are labeled $1_1,1_2\dots ,1_{2N}$, and the nodes of port 2 are labeled $2_1,2_2$; cf. Supplementary Information §2.4. Node $2_1$ is also node $O$. For even $N$, the voltage at $1_1$ and $1_{2N}$ is 0. When $\widetilde{C}_\textrm{t}$ is absent, $1_1$ is not connected to ground. The other half of the equivalent circuit is obtained by reversing the directions of $\widetilde{V}_k$ and $I_\textrm{sh}$, and replacing $O\rightarrow O^\prime$. (e) General full-circuit cascaded-network representation of $N$-gap shielded loops, including the 1-gap in (b), the 2-gap in (c), and the 3-gap in (d). Half is denoted without a prime; the other half is denoted with a prime. The input impedance of the half of $\mathsf{T}$ at port 1, i.e. $Z_{\widetilde{\mathsf{T}}}$ or $Z_{\widetilde{\mathsf{T}}^\prime }$, equals half the impedance of $\mathsf{T}$ at the port where the loop impedance ${Z}_\textrm{sh}$ is connected, i.e. ${\widetilde{Z}}_\mathsf{T}$; the same applies to $Z_{\widetilde{\mathsf{M}}}={\widetilde{Z}}_\mathsf{M}$ and $Z_{\widetilde{\mathsf{A}}}={\widetilde{Z}}_\mathsf{A}$. $1_{2N}$ and $2_2$ are the same node as exemplified in (**b**), (**c**), and (**d**). Disconnecting all blue components does not change $\widetilde{X}_\mathsf{O}$ at $\omega _\textrm{IMax}$. The tuning component $\widetilde{C}_\textrm{t}$, if used, must remain connected because $\widetilde{C}_\textrm{t}$ belongs to $\widetilde{\mathsf{T}}$, as exemplified in (**b**), (**c**), and (**d**). Subfigures (**a**), (**b**) are re-used from Wang et al.^35^.

1. Although a shielded loop *behaves like* a parallel R-L-C circuit with a current source, it *is not* a parallel R-L-C circuit. The proper method to suppress its loop current is by neutralizing the *reactance* of the shielded loop at the output and presenting a low *resistance* to the shielded loop, rather than neutralizing the susceptance of the shielded loop and presenting a high conductance.
2. For a multi-gap shielded loop as shown in Fig. 1, regardless of whether tuning components (e.g., $C_\textrm{t}$ in Fig. 2(a)) are present or whether the gaps are evenly distributed along the shielded loop, the reactance of the shielded loop $X_\mathsf{O}$ at the resonant frequency can be calculated by disconnecting all loop inductors formed by the outer shield. This disconnection does not change the reactance of the shielded loop at the resonant frequency. To completely suppress the loop current, the component $\mathsf{M}$ placed at the output of the shielded loop must have low equivalent series resistance (ESR) and a reactance of $X_\mathsf{M}=-X_\mathsf{O}$.
3. If the shielded loop has no tuning component, or if tuning components are not directly connected to the cable sections containing the output of the shielded loop, then the current-suppression component is or resembles a low-loss inductor, regardless of whether the gaps are evenly distributed.
4. For an $N$-gap shielded loop ($N\ge 1$) with evenly distributed gaps and no tuning components, a low-loss inductor is required to completely suppress the current. Its inductance is ${L}_\textrm{sh}/N$, where ${L}_\textrm{sh}$ is the loop inductance formed by a loop antenna of the same dimensions as the shielded loop. For a wire radius $a$ and a loop radius $b$^1^,
  1 $$\begin{aligned} {L}_\textrm{sh}\approx \mu _0 b\left( \ln {\frac{8b}{a}}-2\right) \,. \end{aligned}$$

All the propositions above are applicable whether a sample is present or absent. However, in the presence of a sample, the loop inductance ${L}_\textrm{sh}$ can deviate significantly from the value given by (1).

Below exemplifies the interpretation of Propositions 1–4.

I. For a single-gap shielded loop where $N = 1$, the propositions are interpreted as follows, whether a sample is present or absent:
  1. The correct approach to suppressing loop current is to neutralize the reactance $X_\mathsf{O}$ and present a low resistance $R_\mathsf{O}$, rather than to neutralize the susceptance $B_\mathsf{O}$ and present a high conductance $G_\mathsf{O}$.
  2. Construct an equivalent circuit as shown in Fig. 2(b) to obtain the output reactance $X_{\mathsf{O}}$. Remove the loop inductor ${L}_\textrm{sh}$ to obtain the output reactance $X_\mathsf{O}^\prime$. At the resonant frequency, $X_\mathsf{O}=X_\mathsf{O}^\prime$. To fully suppress the loop current, the component $\mathsf{M}$ at the loop output must have low ESR and satisfy $X_\mathsf{M} = -X_\mathsf{O}$, regardless of whether tuning components are present. From Propositions 1 and 2, it can be shown that at the resonant frequency $\omega _\textrm{IMax}$ and in the absence of tuning components, $X_\mathsf{O} = - \omega _\textrm{IMax}{L}_\textrm{sh}$ and $X_\mathsf{M} = -X_\mathsf{O} = \omega _\textrm{IMax}{L}_\textrm{sh}$.
  3. In the absence of tuning components, the current-suppression component $\mathsf{M}$ is, or closely resembles, a low-loss inductor.
  4. In the absence of tuning components, a low-loss inductor with inductance ${L}_\textrm{sh} / N = {L}_\textrm{sh}$ is required to fully suppress the loop current, where ${L}_\textrm{sh}$ is given by (1) in the absence of a sample. This is consistent with the analysis of Mollaei et al.^22^. In the presence of a sample, ${L}_\textrm{sh}$ can deviate significantly from the value given by (1).
II. For a three-gap shielded loop where $N = 3$, the propositions are interpreted as follows, whether a sample is present or absent:
  1. The correct approach to suppressing loop current is to neutralize the reactance $X_\mathsf{O}$ and present a low resistance $R_\mathsf{O}$, rather than to neutralize the susceptance $B_\mathsf{O}$ and present a high conductance $G_\mathsf{O}$.
  2. In Fig. 2(d), remove the loop inductor ${L}_\textrm{sh}$ to obtain the output reactance $X_\mathsf{O}^\prime$. At the resonant frequency, $X_\mathsf{O}=X_\mathsf{O}^\prime$. To fully suppress the loop current, the component $\mathsf{M}$ at the loop output must have low ESR and satisfy $X_\mathsf{M} = -X_\mathsf{O}$, regardless of whether tuning components are present. From Propositions 1 and 2, it can be shown that at the resonant frequency $\omega _\textrm{IMax}$, when tuning components do not exist on $\textrm{G}_2$, $X_\mathsf{O} = - 2 Z_0 \cot \left( \beta _\textrm{IMax} l_3 \right)$ and $X_\mathsf{M} = -X_\mathsf{O} = 2 Z_0 \cot \left( \beta _\textrm{IMax} l_3 \right)$. In the complete absence of tuning components, $X_\mathsf{M} = -X_\mathsf{O} = \omega _\textrm{IMax}{L}_\textrm{sh}$.
  3. When tuning components do not exist on $\textrm{G}_2$, the current-suppression component $\mathsf{M}$ is, or closely resembles, a low-loss inductor.
  4. In the absence of tuning components, a low-loss inductor with inductance ${L}_\textrm{sh} / N = {L}_\textrm{sh} / 3$ is required to fully suppress the loop current, where ${L}_\textrm{sh}$ is given by (1) in the absence of a sample. In the presence of a sample, ${L}_\textrm{sh}$ can deviate significantly from the value given by (1).

Proposition 1 facilitates the optimal design of a matching network for minimal loop current. Proposition 2 can be applied to calculate the component values for current suppression while avoiding rapid impedance changes near the resonant frequency of a parallel R-L-C circuit. Propositions 3 and 4 enable the rapid calculation of inductor values for current suppression. A comparison between brute-force calculation and the propositions presented in this article can be found in §1 in the Supplementary Information.

The formal statements are presented in the “Propositions” section within the “Theory” section based on the models in the “Structures and Working Mechanism of Shielded Loops” and “Definitions of Resonance” sections within “Theory”. Their derivation is provided in the “Theory” section and in the Supplementary Information. These propositions are experimentally validated, which requires a calibration method for double-loop probe measurements^25^, of which a brief overview can be found in Section “Calibrating a Double-Loop H-field Probe”.

## Results

**Fig. 3:**
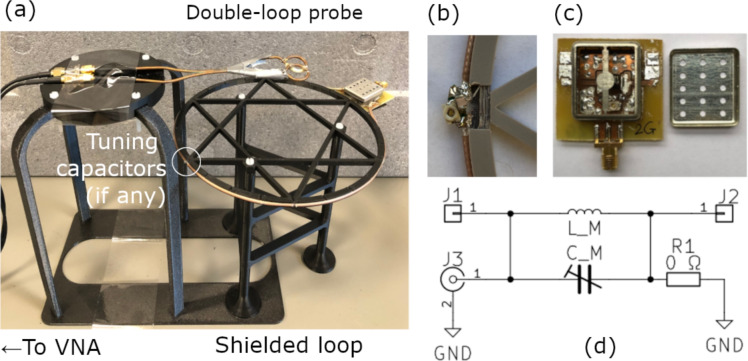
(**a**) Experimental setup. A double-loop probe measures the loop current. The double-loop probe is connected to a VNA. (**b**) Tuning capacitors. (**c**) PCB. An RF shield is installed to suppress the mutual inductance between a loop and the inductor on the board. (**d**) Circuit schematic of (**c**).

**Fig. 4:**
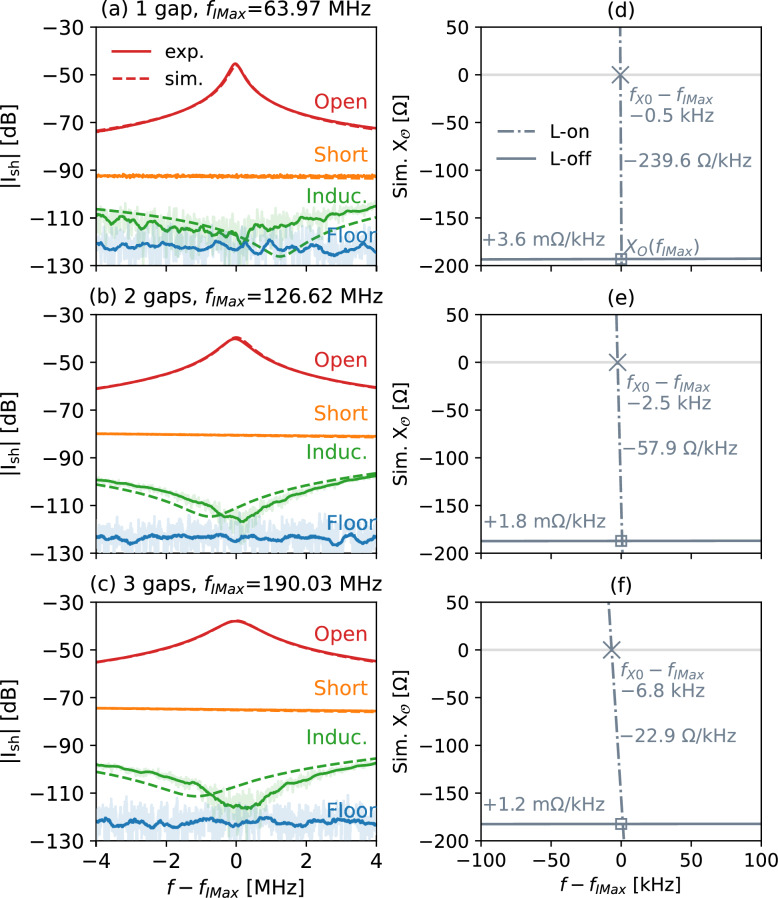
Loop current when $OO^\prime$ are open, shorted, terminated by an inductor or L–C parallel combination (“Induc.”), for (**a**) 1-gap, (**b**) 2-gap, and (**c**) 3-gap shielded loops without any tuning components, and simulated $X_{\mathsf{O}}$ of (**d**) 1-gap, (**e**) 2-gap, and (**f**) 3-gap shielded loops. Experimental $|S_{21}|$ values are converted to shield current by (2). “Floor” is the detection limit of $|S_{21}|$ of the VNA. “Induc.” and “Floor” are smoothed. The original data of “Induc.” and “Floor” are shown translucent. “L-on” means all ${L}_\textrm{sh}$ or ${Z}_\textrm{sh}$ are connected to $\mathsf{T}$ in the simulation setup, as illustrated in Fig. 2. “L-off” means all ${L}_\textrm{sh}$ or ${Z}_\textrm{sh}$ are disconnected from $\mathsf{T}$ in the simulation setup, i.e. all blue components in Fig. 2 are erased. By unplugging all ${L}_\textrm{sh}$, $X_\mathsf{O}$ becomes more numerically stable. The IMax-resonant frequencies $\omega _\textrm{IMax}$ are in the middle of the plots, $f - f_\textrm{IMax} = 0$, as marked by $\square$ and annotated as $X_\mathsf{O}\left( f_\textrm{IMax}\right)$. The X0-resonant frequencies $\omega _\textrm{X0}$ are where “L-on” curves cross $X_\mathsf{O}=0$, as marked by $\times$. In all cases, the IMax-resonant frequencies $\omega _\textrm{IMax}$ are almost the same as X0-resonant frequencies $\omega _\textrm{X0}$. At $\omega _\textrm{IMax}$, the “L-on” curves intersect with the “L-off” curves at the square marker, as stated by Proposition 2: disconnecting all ${L}_\textrm{sh}$’s does not change $X_\mathsf{O}$.

**Fig. 5:**
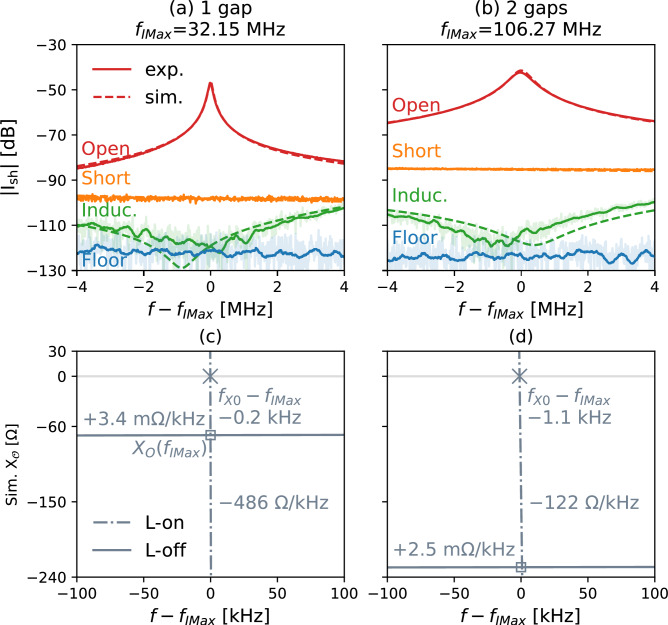
Loop current when antenna outputs $OO^\prime$ are open, shorted, terminated by an inductor or L–C parallel combination, for (**a**) 1-gap and (b) 2-gap shielded loops with a tuning capacitor at $\textrm{G}_0$, as illustrated in Fig. 2, and $X_{\mathsf{O}}$ for (c) 1-gap and (d) 2-gap shielded loops. $|S_{21}|$ values are converted to shield current by (2). “Induc.”, “Floor”, “L-on”, “L-off” mean the same as in Fig. 4. $\omega _\textrm{IMax}$ and $\omega _\textrm{X0}$ are marked in the same way as in Fig. 4. By unplugging all ${L}_\textrm{sh}$, $X_\mathsf{O}$ becomes numerically more stable. In both cases, the IMax-resonant frequencies $\omega _\textrm{IMax}$ are almost the same as X0-resonant frequencies $\omega _\textrm{X0}$. At $\omega =\omega _\textrm{IMax}$, the “L-on” curves intersect with the “L-off” curves, as stated by Proposition 2.

To validate Propositions 1–4, five shielded loops are tested: a 1-gap loop without tuning components, a 2-gap loop without tuning components, a 3-gap loop without tuning components, a 1-gap loop with a tuning capacitor, a 2-gap loop with a tuning capacitor at position $\textrm{G}_0$ (refer to Fig. 1 and Fig. 3(a), (b)). Samples are not used. Inductor–capacitor $L_\mathsf{M}$–$C_\mathsf{M}$ parallel combinations are employed as tunable inductors with the capacitor tuning up the equivalent inductance $L_\textrm{eq}$ as shown in Fig. 3(d)^26^, of which the equivalent inductance $L_\textrm{eq}$ is measured with a vector network analyzer (VNA). Detailed specifications and measurement steps are provided the “Methods” section. The results are in Table 1, Fig. 4, Fig. 5.

**Table 1:** Resonant frequencies (“Rs. Freq.”), nuclei with the closest Larmor frequencies to the resonant frequencies (“Nucleus”), inferred values of inductors $L_\mathsf{M}$ and trimmer capacitors $C_\mathsf{M}$ in experiments, equivalent inductance $L_\textrm{eq}$, inferred loop inductance ${L}_\textrm{sh}$, and theoretical inductance $L_\textrm{te}$. For “no tuning”, $L_\textrm{te}=L_\textrm{sh}/N$. All loop diameters are 16 cm. Calculated ${L}_\textrm{sh}=443.6\ \textrm{nH}$.

$N$	No Tuning			Tuned	
	1	2	3	1	2
$f_\textrm{IMax}$ [MHz]	63.97	126.62	190.03	32.15	106.27
Nucleus	$^{1}$H, 1.5T	$^{1}$H, 3.0T	—	$^{13}$C, 3.0T	$^{23}$Na, 9.4T
$C_\textrm{t}$ [pF]	—	—	—	43.89	9.89
$L_\mathsf{M}$ [nH]	327.01	131.34	99.71	305.47	211.92
$C_\mathsf{M}$ [pF]	2.47	2.93	—	10.89	1.03
$L_\textrm{eq}\left( f_\textrm{IMax}\right)$ [nH]	452.63	235.27	151.71	396.40	331.95
${L}_\textrm{sh}$ [nH]	483.30	471.61	460.46	440.83^†^	445.55^†^
$L_\textrm{te}$ [nH]	483.30	235.81	153.49	375.32	341.89
$X_\textrm{te}\left( f_\textrm{IMax}\right)$ [$\Omega$]	194.26	187.60	183.26	75.82	228.28
Errors $L_\textrm{te}$, $L_\textrm{eq}$	6.8%	0.2%	1.2%	5.3%	3.0%

†: Has no direct relation with $L_\textrm{te}$.

Table 1 presents the resonant frequencies $f_\textrm{IMax}$, the values of tuning capacitors $C_\textrm{t}$ used to tune the 1-gap and the 2-gap shielded loops, the values of the inductors $L_\mathsf{M}$ and the values of the trimmer capacitors $C_\mathsf{M}$ inferred from experiments, the equivalent inductance at the resonant frequency $L_\textrm{eq} \left( f_\textrm{IMax} \right)$ of the $L_\mathsf{M}$–$C_\mathsf{M}$ parallel combination, the theoretical inductance $L_\textrm{te}$ to completely suppress loop current, and the relative difference between $L_\textrm{eq} \left( f_\textrm{IMax} \right)$ and $L_\textrm{te}$.

For 1-, 2- and 3-gap shielded loops without tuning components ($C_\textrm{t}$ is “—”), with a loop diameter 16 cm, and a cable outer diameter 2.10 mm, the loop inductance is calculated as ${L}_\textrm{sh}=443.6\ \textrm{nH}$ according to (1). After accounting for loop length and gap variations in Advanced Design System, it is determined that ${L}_\textrm{sh}=483.30\ \textrm{nH}$ for the 1-gap loop in the experiment, ${L}_\textrm{sh}=471.61\ \textrm{nH}$ for the 2-gap loop in the experiment, and ${L}_\textrm{sh}=460.46\ \textrm{nH}$ for the 3-gap loop in the experiment. For shielded loops with a tuning capacitor $C_\textrm{t}$, Proposition 4—which pertains to shielded loops without tuning components—does not apply, and the loop inductance ${L}_\textrm{sh}$ is not directly related to the theoretical inductance $L_\textrm{te}$ required to suppress the loop current ${I}_\textrm{sh}$.

For 1-, 2-, and 3-gap shielded loops without tuning components ($C_\textrm{t}$ is “—”), according to Proposition 4, $L_\textrm{te}= {L}_\textrm{sh} / N$; for example for the 3-gap loop $N = 3$, so $L_\textrm{te} = {L}_\textrm{sh} / 3 = 153.49~\textrm{nH}$. In theory, for all three cases, the component that completely suppresses the loop current is an inductor. In experiments, as shown in Fig. 4(a)–(c), the $L_\mathsf{M}$–$C_\mathsf{M}$ parallel combinations listed in Table 1 are also equivalent to inductors at the resonance frequencies and suppress loop current to a minimum. This is further demonstrated in Fig. 4(a), (b), (c): installing inductor-equivalent $L_\mathsf{M}$–$C_\mathsf{M}$ parallel combinations at the loop outputs (refer to Fig. 1(b), (c)) results in a significantly greater level of current suppression compared with shorting the outputs, both theoretically and experimentally. Thus Proposition 3 is verified. The theoretical inductance values $L_\textrm{te}={L}_\textrm{sh}/N$ as derived from Proposition 4 are close to the equivalent inductance values $L_\textrm{eq}$ of the L–C parallel combinations used in experiments, with a maximum error of 6.8%. This validates Proposition 4.

Furthermore, as shown in Fig. 4(d), (e), and (f) for 1-, 2-, 3-gap shielded loops without tuning components, the output reactance near resonance typically changes very rapidly (“L-on”), with rates exceeding 22.9 $\Omega$/kHz; after unplugging loop inductors ${L}_\textrm{sh}$’s (“L-off”) in the equivalent circuits Fig. 2(b), (c), and (d), the output reactance $X_\mathsf{O}$ changes only slightly, with rates below 3.6m$\Omega$/kHz. Nevertheless, the “L-on” and “L-off” curves intersect at the resonant frequency $f_\textrm{IMax}$, where $X_\mathsf{O} = -X_\textrm{te}$, as indicated in Table 1. This verifies Proposition 2. This also demonstrates that unplugging inductors in the equivalent circuits makes $X_\mathsf{O}$ significantly less susceptible to steep numerical gradients near the resonant frequency; it can be used as a reliable method to determine the inductance required to suppress loop current. Since Proposition 2 is validated, Proposition 1 is also confirmed.

Furthermore, in Fig. 4(d), (e), and (f) the difference between the X0-resonant frequency $f_\textrm{X0}$ and the IMax-resonant frequency $f_\textrm{IMax}$ is small. For 1-, 2-, 3-gap loops the differences are $0.5~\textrm{kHz}/63.97~\textrm{MHz} = 7.8~\textrm{ppm}$, $2.5~\textrm{kHz}/126.62~\textrm{MHz} = 20~\textrm{ppm}$, $6.8~\textrm{kHz}/190.03~\textrm{MHz} = 36~\textrm{ppm}$, respectively (${1}~\mathrm{ppm} = 1/1~000~000$). Hence in practice X0-resonance and IMax-resonance are practically indistinguishable. However, as demonstrated in the “Theory” section, IMax-resonance provides a theoretical framework through which Propositions 1–4 can be derived.

For shielded loops with a tuning capacitor $C_\textrm{t}$ at position $\textrm{G}_0$, as written in Table 1, the 1-gap ($N = 1$) shielded loop is tuned to 32.15MHz by $C_\textrm{t} = {43.89}\textrm{pF}$, and the 2-gap ($N = 2$) shielded loop is tuned to 106.27MHz by $C_\textrm{t} = {9.89}\textrm{pF}$. The loop current measurements are in Fig. 5. In the case of the 1-gap shielded loop, the tuning capacitor $C_\textrm{t}$ is directly connected to the cable sections containing the antenna output, which is represented as $\textrm{T}_1$ in Fig. 2(b), so Proposition 3 does not apply. In the case of the 2-gap shielded loop, the tuning capacitor $C_\textrm{t}$ is not directly connected to the cable sections containing the antenna output represented as $l_2$ in Fig. 2(c), so Proposition 3 applies. In this case the component required to completely suppress the loop current ${I}_\textrm{sh}$ is an inductor, as predicted by Proposition 3; thus Proposition 3 is validated for the 2-gap shielded loop with a tuning capacitor.

However, Propositions 1 and 2 are applicable to all shielded loops near fundamental resonance. As shown in Fig. 5(b), (d) for 1-, 2-gap shielded loops with $C_\textrm{t}$, the output reactance near resonance typically changes faster than 122 $\Omega$/kHz (“L-on”); after unplugging loop inductors ${L}_\textrm{sh}$’s (“L-off”) in the equivalent circuits shown in Fig. 2(b), (c), the output reactance $X_\mathsf{O}$ changes more slowly than 3.4 m$\Omega$/kHz. The “L-on” and “L-off” curves intersect at the resonant frequency $f_\textrm{IMax}$ where $X_\mathsf{O} = -X_\textrm{te}$ as written in Table 1. This verifies Proposition 2. Again, for shielded loops with tuning components, unplugging inductors in the equivalent circuits makes $X_\mathsf{O}$ significantly less susceptible to steep numerical gradients near the resonant frequency; it can be used as a reliable method to determine the inductance needed to suppress loop current. Since Proposition 2 is verified for shielded loops with tuning components, Proposition 1 is also true.

The difference between X0-resonance and IMax-resonance is $0.2~\textrm{kHz}/32.15~\textrm{MHz} = 6.2~\textrm{ppm}$ for the 1-gap tuned shielded loop, and $2.5~\textrm{kHz}/126.62~\textrm{MHz} = 20~\textrm{ppm}$ for the 2-gap tuned shielded loop.

In all the cases experimentally validated, whether with or without tuning components, as shown in Fig. 4 and Fig. 5(a), (b), by installing inductors at antenna outputs $OO^\prime$, the loop current amplitude $|{I}_\textrm{sh}|$ is reduced by an additional 31dB to 36dB. Clearly, for suppressing the loop current ${I}_\textrm{sh}$, shorting the antenna outputs is not optimal.

## Discussion

Table 1 shows fitted ${L}_\textrm{sh}$ values vary around ${L}_\textrm{sh}={443.6}\textrm{nH}$. This is likely because of errors in coil construction. The coaxial cable is severed at the loop output and bridged by copper filling zones of printed circuit boards (PCBs), resulting in a loop that is not perfectly circular. The gap sizes, gap positions, and the contacts between coaxial cables and PCBs vary. The simulations cannot fully account for these factors, so ${L}_\textrm{sh}$ values determined from experimental data vary.

During simulation, propagation loss is modeled into coaxial cables as $\tan \delta$ and conductor resistivity. However, in our work, propagation loss can also be neglected. The loop perimeter is 0.50m, the cables are RG-316, and, referring to Supplementary Table S1, the wave propagation loss is at most 0.09dB at 50MHz, 0.13dB at 100MHz, and 0.20dB at 200MHz. Had the shielded loop been constructed from RG-178 or thinner cables, the losses would have increased and might no longer be negligible.

In the experiments, samples are not used so that the loop inductance is not affected. In practical MRI, samples are present near shielded loops. This will change loop inductance ${L}_\textrm{sh}$. Notwithstanding, Propositions 1–4 still apply. Although it is difficult to know ${L}_\textrm{sh}$ in advance, the change of $L_\mathsf{M}$ is mild near resonance, as exemplified in Fig. 4(d), (e), and (f) and Fig. 5(c), (d). This means that in practice substituting sample-free ${L}_\textrm{sh}$ in $L_\mathsf{M}={L}_\textrm{sh}/N$ provides a good starting point for fine-tuning $L_\mathsf{M}$ to achieve minimum $\left| {I}_\textrm{sh}\right|$ at $\omega _\textrm{IMax}$.

As Jabbari et al.^27^ note, mutual inductance can exist between different loop segments ${L}_{\textrm{sh},k}$. In this article, however, mutual inductance is not incorporated into the theoretical analysis or simulation modeling. This has not led to significant errors. As Table 1 indicates, the errors between the theoretical inductance values and the actual inductance values are below 7% for the coils at fundamental resonance. As frequency increases and the coil enters the second resonance, it may become necessary to incorporate the inter-segment mutual inductance.

“High-impedance coils” can be made from other types of cables such as twisted pairs^28^ and striplines^27^. A stripline-based shielded loop has a similar structure to a coaxial shielded loop. In this case Propositions 1–4 are applicable. Twisted pairs, however, have different working mechanisms as both wires are exposed to the external magnetic field and contribute to the loop current. In this case Propositions 1–4 may not apply. Moreover, shielded loops can be made resonant when the antenna outputs are short-circuited^29^. In this case Propositions 1–4 may not apply.

## Conclusion

Systematic methods to determine the termination for suppressing the loop current of shielded loops at fundamental resonance are presented: introduce a component that cancels the antenna reactance; unplug loop inductors to determine the antenna reactance; and, when no tuning components are present and all gaps are evenly distributed, use an inductive component with an inductance of ${L}_\textrm{sh}/N$. The difference between shorting antenna outputs and maximal current suppression is also confirmed experimentally. These results can assist designers in developing loop arrays that produce higher-quality images.

## Methods

### Materials

Five RG-316 coaxial cables are made into five shielded loops with a diameter of 16 cm: a 1-, a 2-, and a 3-gap loop without tuning components, and a 1- and a 2-gap loop with tuning capacitors $C_\textrm{t}$. The experimental setup is shown in Fig. 3. Tuning capacitors are installed at $\textrm{G}_0$, as shown in Fig. 2(a)–(d) and Fig. 3(a), (b). The resonant frequencies of these shielded loops are measured using a custom-built double-loop H-field probe connected to a Rohde & Schwarz ZNL3 vector network analyzer (VNA) (Rohde & Schwarz; Munich, Germany). Printed circuited boards (PCBs) are fabricated with FR-4. Coilcraft air-core inductors (Coilcraft Inc.; Cary, IL, USA) are used to suppress loop current. For the 1- and 2-gap shielded loops with tuning capacitors $C_\textrm{t}$, low-ESR fixed-value (Passive Plus Inc.; Huntington, NY 11743, USA) and trimmer capacitors (Knowles Electronics; Itasca, IL 60143, USA) are used at gap $\textrm{G}_0$ to tune the shielded loops. For all shielded loops, inductors $L_\mathsf{M}$ are installed at the loop outputs, as shown in Fig. 1(c) and Fig. 2(a)–(d), to suppress the loop current. In some cases, inductor-trimmer $L_\mathsf{M}$–$C_\mathsf{M}$ parallel combinations as shown in Fig. 3(d) are used as tunable inductors with the capacitors $C_\mathsf{M}$ increasing the equivalent inductance $L_\textrm{eq}$ of the parallel combination^26^. The frequency of maximum current suppression decreases with increasing equivalent inductance. After each loop current measurement, a VNA is used to measure the equivalent inductance $L_\textrm{eq}$ of the parallel inductor-capacitor combination $L_\mathsf{M}$–$C_\mathsf{M}$. To eliminate the influence of mutual inductance between oSo of the shielded loops and the inductors $L_\mathsf{M}$ on the PCBs, RF shields (Würth Elektronik; 74638 Waldenburg, Germany) are installed on PCBs, as shown in Fig. 3(c).

No samples are used in the experiment for two reasons. First, samples can significantly alter the loop inductance in the frequency range of interest, making it challenging to model in circuit simulations. Second, samples modify the current sensing path of a double-loop H-field probe^25^, causing the calibrated $S_{21}$ of a double-loop H-field probe to deviate substantially from $\left| S_{21}\right| \approx \omega \kappa \left| {I}_\textrm{sh} \right|$, thereby introducing additional errors in loop current measurements.

### Experiments

The circuit simulator is Advanced Design System (ADS) (Keysight Technologies; Santa Rosa, CA, USA). In simulations the coaxial cable’s parameters are taken from the standard specifications of RG-316 coaxial cables: iC’s diameter 0.454 mm, oSi’s diameter 1.52 mm, $\epsilon _r=2.1$, $\tan \delta = 0.002$. The conductor resistivity is $1.340\times$ copper resistivity to account for outer shield braiding. The experiment for a shielded loop is performed as follows: (1) If there is no tuning capacitor, measure the resonance directly; otherwise install fixed-value and trimmer capacitors at $\textrm{G}_0$, keep the coil output open-circuited, and tune the resonance to the desired frequency; (2) Calibrate the double-loop H-field probe following the method described in Section “Calibrating a Double-loop H-field Probe”; (3) Install $L_\mathsf{M}$, $C_\mathsf{M}$ and an RF shield onto the PCB and tune for maximum current suppression at the resonant frequency, with the $L_\mathsf{M}$ and $C_\mathsf{M}$ values determined according to Propositions 1–4; (4) Remove the PCB from the shielded loop, install the 0$\Omega$ jumper R1, and measure the equivalent impedance using a VNA at J3 as shown in Fig. 3(d); (5) Determine the inductor $L_\mathsf{M}$ and the capacitor $C_\mathsf{M}$ values from the measurements.

### Calibrating a double-loop H-field probe

A calibration method^25^ is employed to correct for residual probe coupling:

1. Connect a probe to a VNA. Tighten all connections. Secure the probe position. Ensure that all shielded loops remain in the same place throughout all measurements.
2. Remove the shielded loop. Record the $S_{21}$ measurement results as $S_{21,a}$.
3. Put back the shielded loop. Record the $S_{21}$ measurement results as $S_{21,b}$.
4. Take the complex-number difference $S_{21,\text {cal}}=S_{21,b}-S_{21,a}$.

There is $\left| S_{21,\text {cal}}\right| \approx \kappa \omega \left| {I}_\textrm{sh}\right|$, where $\kappa$ is a constant related to the specifics of a setup. The exact value value of $\kappa$ need not be known^25^. In our experiments $\left| S_{21,\text {cal}}\right| \times \omega _\textrm{IMax}/\omega$ is used as the magnitude of measured loop current relative to that at $\omega _\textrm{IMax}$. This quantity is compared against simulation. In decibels the expression is

2 $$\begin{aligned} \textrm{dB}\left[ {I}_\textrm{sh}\right] =\textrm{dB}\left[ S_{21,\text {cal}}\right] -20\lg {\omega }+20\lg {\omega _\textrm{IMax}}\,. \end{aligned}$$

## Theory

### Structure and working mechanism of shielded loops

In this article, only axially symmetric shielded loops made of coaxial cables are addressed. Typically, such an $N-$gap shielded loop is formed by alternating outer gaps and inner gaps. At an outer gap, outer conductors are cut while inner conductors join. At an inner gap, outer conductors join while inner conductors are cut. The gaps can be unevenly or evenly distributed. Evenly distributed gaps are spaced every $180^\circ /N$. These gaps are denoted as $\textrm{G}_{k}$ and $\textrm{G}_{k^\prime }$, $k=0,1,\dots ,N-1$. In practice, a tuning component can also be placed at gap $\textrm{G}_0$^30^. Shielded loops of $N=1,2,3$ gaps are shown in Fig. 1(c). The output port is located midway, where outer conductors join and inner conductors are exposed. The two nodes of the exposed inner conductors are labelled as $O$ and $O^\prime$. The antenna output port $OO^\prime$ can be connected to a network $\mathsf{M}$. The impedance looking toward $\mathsf{M}$ at port $OO^\prime$ is denoted as $Z_\mathsf{M}$. An amplifier $\mathsf{A}$ with an input impedance $Z_\mathsf{A}$ where ${{\,\mathrm{\textrm{Re}}\,}}Z_\mathsf{A}>0$ and $\left| Z_\mathsf{A}\right| <\infty$ can be further connected to $\mathsf{M}$. $\mathsf{M}$ presents impedance $Z_{\textrm{o}\mathsf{M}}$ to amplifier $\mathsf{A}$. Usually, $Z_{\textrm{o}\mathsf{M}}$ is chosen to be the optimal input impedance of amplifier $\mathsf{A}$. Other values may be used if required. The cross-section of a shielded loop, shown in Fig. 1(a), consists of three parts: the inner conductor iC, the inner surface of the outer shield oSi, and the outer surface of the outer shield oSo.

The following are assumed regarding the working mechanism of a shielded loop:

(i) The frequency is sufficiently high that the skin depth^31^ is far shallower than the shield thickness, so oSi and oSo can be deemed independent.
(ii) Each loop segment is no longer than 0.1 wavelengths in air so the current on oSo can be deemed constant.
(iii) Following assumption (ii), the loop antenna formed by oSo can be well approximated by an inductor.
(iv) Each transmission line is shorter than 1/4 wavelength in the dielectric.
(v) All transmission lines are in TEM mode.
(vi) The coaxial cable of which the shielded loop is made has negligible losses.
(vii) Network $\mathsf{M}$ has negligible losses.

For a typical RG-316 coaxial cable, the thickness of the outer shield is about 0.25 mm. Above 2.44 MHz, the skin depth is less than 1/6 of shield thickness. Most MRI applications are above this frequency, and Assumption (i) holds. Assumptions (ii) and (iii) are typical requirements for loop antennas/coils in MRI^32^, as uneven current distribution will induce electric dipole modes^1^ and degrade the signal-to-noise ratio (SNR) of signal reception. Whether Assumption (vi) holds depends on the cable type, frequency, and length; for example, the attenuation a typical RG-316 coaxial cable is $\sim 0.55~\mathrm {dB/m}$ at $400~\textrm{MHz}$ and $\sim 0.26~\mathrm {dB/m}$ at $100~\textrm{MHz}$, and that of a typical RG-178 cable is $\sim 0.95~\mathrm {dB/m}$ at $400~\textrm{MHz}$ and $\sim 0.46~\mathrm {dB/m}$ at $100~\textrm{MHz}$, as shown in Supplementary Table S1. Cables thinner than RG-178, such as Huber+Suhner K_01152 (Herisau, Appenzell Ausserrhoden, Switzerland), can exhibit losses as high as $1.5~\mathrm {dB/m}$. Since cable loss degrades SNR, coil designers should minimize cable loss to ensure Assumption (vi) remains valid. Similarly, since matching network loss also degrades SNR, designers should minimize matching network loss to ensure Assumption (iv) remains valid. However, the criterion for “low loss” varies by application; therefore, a universal threshold cannot be specified.

When a shielded loop receives an electromagnetic wave, voltages are induced at the gaps $\textrm{G}_{k}$ and $\textrm{G}_{k^\prime }$, $0\le k\le N-1$, which subsequently generate alternating currents on the oSo segments. These current branches, referred to as “loop current” and denoted as ${I}_{\textrm{sh},k}$ and ${I}_{\textrm{sh},k^\prime }$, $1\le k\le N$, flow into coaxial cable sections, and then to the electrical load $\mathsf{M}$. On oSo, gap voltages can be modeled as voltage sources in series with loop antenna/coil segments that combine to form a loop with the same dimensions as the shielded loop^8^, as shown in Fig. 1(c). For MRI applications, the loop can be well approximated by a lossy inductor. Denote the inductance as ${L}_\textrm{sh}$ and the resistance as ${R}_\textrm{sh}$. Following this model, the equivalent circuits of a 1-gap shielded loop can be drawn as shown in Fig. 2(a). This is the same model as that in Jabbari et al.^27^ At the output port of the shielded loop, $OO^\prime$, the shielded loop presents impedance $Z_\mathsf{O}$ to its electrical load. Since the shielded loop is reflectionally symmetric, the equivalent half circuit can be drawn as Fig. 2(b). The equivalent half circuits of 2- and 3-gap shielded loops can be drawn as Fig. 2(c) and (d) following the same procedure.

### Definitions of resonance

Assumption (ii) implies the current is constant on oSo:

3 $$\begin{aligned} I_{\textrm{sh},1} = I_{\textrm{sh},1^\prime } = I_{\textrm{sh},2} = I_{\textrm{sh},2^\prime } = \dots = I_{\textrm{sh},N} = I_{\textrm{sh},N^\prime } \end{aligned}$$

due to charge conservation at each gap. Henceforth they are all denoted as ${I}_\textrm{sh}$.

Resonance of shielded loops is defined as in Definition 1.

#### Definition 1

*(IMax-resonant)* A shielded loop is IMax-resonant if, at angular frequency $\omega _\textrm{IMax}$, with the port $OO^\prime$ is open-circuited, i.e. $Z_\mathsf{M}=\infty$, $\left| {I}_\textrm{sh}\right|$ reaches its maximum possible value. $\omega _\textrm{IMax}$ is called the “IMax-resonant frequency”, or “resonant frequency” for short.

As revealed by Lemma 2 in the “Derivation” section, our definition of resonance is equivalent to that used by Nohava et al.^9,13^

Some literature defines resonance as the condition in which the output reactance or susceptance of the shielded loop at port $OO^\prime$is zero^5,22^. This is formally stated in Definition 2.

#### Definition 2

*(X0-resonant)* A shielded loop is X0-resonant if, at angular frequency $\omega _\textrm{X0}$, $X_{\mathsf{O}}=0$ or equivalently $B_{\mathsf{O}}=0$. $\omega _\textrm{X0}$ is called the “X0-resonant frequency”.

Near $\omega _\textrm{X0}$, an $N$-gap shielded loop behaves similarly to a parallel R-L-C circuit^5^, where high impedance is created at port $OO^\prime$. An $N$-gap shielded loop is sometimes referred to as a “high-impedance coil”^5,12,22^.

Our definition of coil resonance typically yields an $\omega _\textrm{IMax}$ that is nearly identical to $\omega _\textrm{X0}$, as shown in Fig. 4 and Fig. 5: $\left| f_\textrm{X0} - f_\textrm{IMax} \right| < 10~\textrm{kHz}$ while $f_\textrm{IMax}> 32~\textrm{MHz}$. Their relative difference is below 40 parts per million (ppm). Thus the current distributions at the two frequencies are virtually the same, an example of which is given in Supplementary Fig. S1 of a one-gap $\varnothing {16}\textrm{cm}$ shielded loop made of RG-316 coaxial cable with no tuning components. From a purely practical standpoint, there is no need to distinguish between these two definitions of resonance. However, Definition 1 ($\omega _\textrm{IMax}$) allows direct measurement of the resonance using a double-loop H-field probe^25^, and it also provides a theoretical framework that directly supports all Propositions 1–4, whereas Definition 2 ($\omega _\textrm{X0}$), though numerically close, does not.

### Propositions

The propositions in Section “Introduction” can be restated rigorously as follows. For an $N$-gap IMax-resonant shielded loop, to fully suppress the loop current ${I}_\textrm{sh}$ at $\omega _\textrm{IMax}$,

#### Proposition 1

$R_\mathsf{M}$ is low and positive, and $X_\mathsf{M}=-X_\mathsf{O}$.

#### Proposition 2

Whether or not tuning components exist, $X_\mathsf{O}$ does not change when ${Z}_\textrm{sh}\rightarrow \infty$, i.e. $X_\mathsf{O}$ does not change when the loop inductors ${L}_\textrm{sh}$ are disconnected from the circuit.

#### Proposition 3

When tuning components are not directly connected to $\textrm{T}_N$ or $\textrm{T}_{N^\prime }$, $X_\mathsf{M}=-X_\mathsf{O}>0\,$. This means $Z_\mathsf{M}$ resembles an inductor.

#### Proposition 4

When the shielded loop has no tuning components, and all $\textrm{T}_k$’s and $\textrm{T}_{k^\prime }$’s have the same length, $Z_\mathsf{M}$ resembles a low-loss inductor of which $L_\mathsf{M}={L}_\textrm{sh}/N$.

The meaning of “low” in Proposition 1 depends on the context:

I. When a shielded loop is terminated solely by components intended for complete suppression of ${I}_\textrm{sh}$, e.g., for current blocking during the transmit phase, low $R_\mathsf{M}$ means all components have high Q or low ESR. The lower the ESR, the lower the resulting $\left| {I}_\textrm{sh}\right|$. In this case, at $\omega _\textrm{IMax}$,
  4 $$\begin{aligned} Z_\mathsf{T}\left( Z_\mathsf{M}\right) =\dfrac{R_\mathsf{O} {R}_\textrm{sh}}{R_\mathsf{M}}-\textrm{j}{X}_\textrm{sh}\, , \end{aligned}$$
  where $Z_\mathsf{T}\left( Z_\mathsf{M}\right)$ denotes the functional dependence of $Z_\mathsf{T}$ on $Z_\mathsf{M}$.
II. When a shielded loop is terminated by a matching network $\mathsf{M}$ that is connected to an amplifier $\mathsf{A}$, low $R_\mathsf{M}$ means all components in $\mathsf{M}$ have low ESR and are tuned such that $X_\mathsf{M}=-X_\mathsf{O}$ and $R_\mathsf{M} < R_\mathsf{O}$. In this case, $R_\mathsf{M}$ cannot be arbitrarily low but is bounded by $Z_{\textrm{o}\mathsf{M}}$, $Z_\mathsf{A}$, and $Z_\mathsf{O}$^21^:
  5 $$\begin{aligned} R_\mathsf{M}=R_\mathsf{O}/\textrm{SWR}\, \end{aligned}$$
  where $\textrm{SWR}={\left( 1+\left| \Gamma _\mathsf{A}\right| \right) }/{\left( 1-\left| \Gamma _\mathsf{A}\right| \right) }$, and $\Gamma _{\mathsf{A}} = \left( Z_{\textrm{o}\mathsf{M}}-Z_\mathsf{A}^*\right) / \left( Z_{\textrm{o}\mathsf{M}}+Z_\mathsf{A}\right)$ is the power wave reflection coefficient^33,34^. Accordingly,
  6 $$\begin{aligned} Z_\mathsf{T}\left( Z_\mathsf{M}\right) ={R}_\textrm{sh}\cdot \textrm{SWR}-\textrm{j}{X}_\textrm{sh}\,. \end{aligned}$$

### Proof of the propositions

Refer to the Supplementary Information for the derivation of Propositions 1–4.

## Supplementary Information

Supplementary Information.

## Data Availability

The datasets used and/or analysed during the current study are available from the corresponding author on reasonable request.
