# Supplementary material for: Suppressing loop current of shielded loops at fundamental resonance
Source: Sci Rep. 2026 Feb 11;16:8400. doi: 10.1038/s41598-026-36956-7 (PMC12972106; doi:10.1038/s41598-026-36956-7)
Supplement: Supplementary file 1 — Supplementary Information. [file 41598_2026_36956_MOESM1_ESM.pdf]

Supplementary Information for Suppressing Loop Current of Shielded Loop antennas on Fundamental Resonance

1 Comparison between direct calculation and Propositions 1–4

For a *one-turn one-gap* shielded loop *in the absence of tuning components*, direct calculation of $\widetilde{Z}_{\mathsf{M}}$ that minimizes $|I_{\mathrm{sh}}|$ is straightforward. However, the addition of a tuning component or multiple gaps (two or more) renders direct calculation extremely cumbersome. Our theoretical framework circumvents these complications, enabling straightforward calculation of the impedance needed for complete suppression of the loop current at fundamental resonance.

To illustrate the advantage of our approach, we consider a 3-gap shielded loop with a tuning capacitor $\widetilde{C}_{\mathrm{t}}$, as depicted in Figure 2(d).

1.1 Direct calculation of $\widetilde{Z}_{\mathsf{M}}$

The direct calculation approach requires performing the following three tasks in sequence:

1. Express I_{sh} in terms of $\left\{\widetilde{V}_k\right\}$, $\widetilde{Z}_{\mathsf{M}}$, $\left\{\widetilde{Z}_{\mathrm{sh},k}\right\}$, $\widetilde{C}_{\mathrm{t}}$, l_1, l_2, l_3, Z_0, β.

2. Determine ω_{IMax}.

3. Find the value of $\widetilde{X}_{\mathsf{M}}$ that minimizes $|I_{\mathrm{sh}}|$ for fixed $\left\{\widetilde{V}_k\right\}$, $\widetilde{Z}_{\mathsf{M}}$, $\left\{\widetilde{Z}_{\mathrm{sh},k}\right\}$, $\widetilde{C}_{\mathrm{t}}$, l_1, l_2, l_3, Z_0, β.

This requires solving three equation sets. Half of the equivalent circuit is illustrated in Supplementary Fig. S2. For transmission line T_1 with the tuning capacitor $\widetilde{C}_{\mathrm{t}}$:

$$\begin{cases} U_1(0) = -U[1_1] = U_{01}^{+} + U_{01}^{-} = \dfrac{I_{\mathrm{t}}}{\mathrm{j}\omega\widetilde{C}_{\mathrm{t}}} \\ U_1(-l_1) = U_{01}^{+}\mathrm{e}^{\mathrm{j}\beta l_1} + U_{01}^{-}\mathrm{e}^{-\mathrm{j}\beta l_1} \\ I_1(0) = \dfrac{U_{01}^{+}}{Z_0} - \dfrac{U_{01}^{-}}{Z_0} = I_{\mathrm{t}} - I_{\mathrm{sh}} \\ I_1(-l_1) = \dfrac{U_{01}^{+}}{Z_0}\mathrm{e}^{\mathrm{j}\beta l_1} - \dfrac{U_{01}^{-}}{Z_0}\mathrm{e}^{-\mathrm{j}\beta l_1} = 0 \\ \widetilde{U}[1_1] + \widetilde{V}_1 = I_{\mathrm{sh}}\widetilde{Z}_{\mathrm{sh},1} \end{cases} \tag{S1}$$

For transmission line T_2:

$$I_{\mathrm{sh}}\left(\widetilde{Z}_{\mathrm{sh},2} + \frac{1}{\mathrm{j}Y_0\tan\beta l_2}\right) = \widetilde{V}_2 \tag{S2}$$

For transmission line T_3 with the $\widetilde{Z}_{\mathsf{M}}$ termination:

$$\begin{cases} U_3(0) = U_{\mathrm{iC3}}(0) - U_{\mathrm{oSi3}}(0) = U_{03}^{+} + U_{03}^{-} \\ U_3(-l_3) = I_{\mathsf{M}}\widetilde{Z}_{\mathsf{M}} = U_{03}^{+}\mathrm{e}^{\mathrm{j}\beta l_3} + U_{03}^{-}\mathrm{e}^{-\mathrm{j}\beta l_3} \\ I_3(0) = -I_{\mathrm{sh}} = \dfrac{U_{03}^{+}}{Z_0} - \dfrac{U_{03}^{-}}{Z_0} \\ I_3(-l_3) = -I_{\mathsf{M}} = \dfrac{U_{03}^{+}}{Z_0}\mathrm{e}^{\mathrm{j}\beta l_3} - \dfrac{U_{03}^{-}}{Z_0}\mathrm{e}^{-\mathrm{j}\beta l_3} \\ I_{\mathrm{sh}}\widetilde{Z}_{\mathrm{sh},3} = \widetilde{V}_3 + U_{\mathrm{oSi3}}(0) = \widetilde{V}_3 - U_3(0) + U_{\mathrm{iC3}}(0) \\ U_{\mathrm{iC3}}(0) = 0 \end{cases} \tag{S3}$$

The notation extends that defined in Supplementary Information section 2.3:

- $I_k(x)$—current in transmission line T_k at position x where $x=0$ is the leftmost point; pin current direction is leftward on iC_k.
- $U_{\mathrm{iC}_k}(x)$—voltage of inner conductor iC_k at position x.
- $U_{\mathrm{oSi}_k}(x)$—voltage of outer shield inner surface oSi_k at position x.
- U_{0k}^{+}, U_{0k}^{-}—forward- and backward-propagating wave voltages in T_k.
- $U_k(x)=U_{\mathrm{iC}_k}(x)-U_{\mathrm{oSi}_k}(x)$—the transmission-line voltage across T_k.

Setting $U_{\mathrm{iC3}}(0)=0$ follows from the claim in section 2.5: although oSo_k and iC_k float in Fig. S3(c), (d), their voltages $U[\mathrm{oSo}_k]$ and $U[\mathrm{iC}_k]$ may be chosen as zero.

Solving these equations yields the result for task 1:

$$\frac{I_{\mathrm{sh}}}{\widetilde{V}_1+\widetilde{V}_2+\widetilde{V}_3}=\left[\frac{\mathrm{j}\omega\widetilde{C}_{\mathrm{t}}Z_0\widetilde{Z}_{\mathrm{sh},1}\cos(\beta l_1)+Z_0\cos(\beta l_1)+\mathrm{j}\widetilde{Z}_{\mathrm{sh},1}\sin(\beta l_1)}{\mathrm{j}\omega\widetilde{C}_{\mathrm{t}}Z_0\cos(\beta l_1)+\mathrm{j}\sin(\beta l_1)}+\widetilde{Z}_{\mathrm{sh},2}-\mathrm{j}Z_0\cot(\beta l_2)\right.$$
$$\left.+\frac{\left(Z_0^2+\widetilde{Z}_{\mathrm{M}}\widetilde{Z}_{\mathrm{sh},3}\right)\cdot\mathrm{j}\sin(\beta l_3)+\left(Z_0\widetilde{Z}_{\mathrm{M}}+Z_0\widetilde{Z}_{\mathrm{sh},3}\right)\cos(\beta l_3)}{Z_0\cos(\beta l_3)+\widetilde{Z}_{\mathrm{M}}\cdot\mathrm{j}\sin(\beta l_3)}\right]^{-1}. \tag{S4}$$

This expression, while exact, applies only to the specific configuration in Fig. 2(d) and provides limited insight into the underlying physics. Moreover, to answer tasks 2 and 3, complicated symbolic calculations must still be performed.

1.2 Using Propositions

Our framework dramatically simplifies this calculation. Proposition 2 states that all gap impedances $\left\{\widetilde{Z}_{\mathrm{sh},k}\right\}$ can be disconnected without affecting the output reactance at ω_{IMax}. Applying this simplification to Fig. 2(d) or Supplementary Fig. S2, the output impedance at ω_{IMax} immediately reduces to

$$\widetilde{Z}_{\mathrm{O}}=-\mathrm{j}Z_0\cot(\beta_{\mathrm{IMax}}l_3)\,. \tag{S5}$$

Proposition 1 then directly gives the matching reactance required to minimize $|I_{\mathrm{sh}}|$:

$$\widetilde{X}_{\mathrm{M}}=-\widetilde{X}_{\mathrm{O}}=Z_0\cot(\beta_{\mathrm{IMax}}l_3)\,, \tag{S6}$$

with no need to solve equation sets (S1)–(S3) or perform algebraic reduction.

Eq. S6 can be calculated directly from measurements or existing data. The characteristic impedance Z_0 and length l_3 are known from the coaxial cable specifications and physical construction. The propagation constant at resonance, $\mathrm{j}\beta_{\mathrm{IMax}}=\mathrm{j}\omega_{\mathrm{IMax}}\cdot\sqrt{\varepsilon_r\mu_r}/c$, can be calculated from the measured resonance frequency ω_{IMax} (obtained via VNA) and the cable's dielectric properties. The relative permittivity ε_r and permeability μ_r are available in cable datasheets, either directly or through related parameters such as velocity factor, signal delay, or capacitance per unit length.

2 Proof of the propositions

2.1 Definitions and Propositions Revisited

Definition 1 (IMax-resonant)**.** *A shielded loop is IMax-resonant if, at angular frequency ω_{IMax}, with the port OO' is open-circuited, i.e. $Z_{\mathrm{M}}=\infty$, $|I_{\mathrm{sh}}|$ reaches its maximum possible value. ω_{IMax} is referred to as the "IMax-resonant frequency", or "resonant frequency" for short.*

Definition 2 (X0-resonant)**.** *A shielded loop is X0-resonant if, at angular frequency ω_{X0}, $X_{\mathrm{O}}=0$ or equivalently $B_{\mathrm{O}}=0$. ω_{X0} is referred to as the "X0-resonant frequency".*

For an N-gap IMax-resonant shielded loop, to fully suppress the loop current I_{sh} at ω_{IMax},

Proposition 1. *R_{M} is low and positive, and $X_{\mathrm{M}}=-X_{\mathrm{O}}$.*

Proposition 2. *Whether or not tuning components exist, X_{O} does not change when $Z_{\mathrm{sh}}\to\infty$, i.e., X_{O} does not change when the loop inductors L_{sh} are disconnected from the circuit.*

Proposition 3. *When tuning components are not directly connected to T_N or $\mathsf{T}_{N'}$, $X_{\mathrm{M}}=-X_{\mathrm{O}}>0$. This means Z_{M} resembles an inductor.*

Proposition 4. *When the shielded loop has no tuning components, and all T_k's and $\mathsf{T}_{k'}$'s have the same length, Z_{M} resembles a low-loss inductor of which $L_{\mathrm{M}}=L_{\mathrm{sh}}/N$.*

2.2 Structure of Theory and Nomenclature

In this section Propositions 1–4 are derived. The propositions that appear in the section "Introduction" are rephrased more rigorously in the "Propositions" and "Proof of the propositions" sections. They are the same propositions as in section "Introduction", expressed differently.

Definition 1 of "IMax-resonant frequency" and Definition 2 of "X0-resonant frequency" are introduced, but only Definition 1 is used for derivation.

The relations between the lemmas, the theorems and the propositions are:

- Lemma 1 is the foundation of the entire derivation.
- Lemmas 2, 3 lead to Theorem 4.
- Theorem 4 leads to Propositions 1–3.
- Lemmas 2, 3 and Theorem 4 lead to Proposition 4.

2.3 Notations

Below is a list of all notations. The order of notations is: numbers → Latin alphabet → Greek alphabet. Capital letters precede minuscule letters.

- 1_k, 2_k—port nodes as shown in Fig. 2.
- A—the preamplifier (low-noise amplifier), i.e., the first-stage amplifier of the receiving chain.
- a—the wire radius of a shielded loop, as annotated in Fig. 1; not used in this section.
- B_{O}—the susceptance at port OO' looking toward the shielded loop, as illustrated in Fig. 2.
- b—the loop radius of a shielded loop, as annotated in Fig. 1; not used in this section.
- C_{M}—the capacitor to tune the impedance of the parallel combination of L_{M}–C_{M}; in this case the preamplifier A is not connected. Not used in this section.
- C_{t}—the tuning capacitors on shielded loops.
- f_{IMax}—the IMax-resonant frequency as defined in Definition 1; $f_{\mathrm{IMax}} = \omega_{\mathrm{IMax}}/(2\pi)$.
- f_{X0}—the X0-resonant frequency as defined in Definition 2; $f_{\mathrm{X0}} = \omega_{\mathrm{X0}}/(2\pi)$.
- G_k, $\mathrm{G}_{k'}$—the k-th and k'-th inner or outer gap. Gaps G_k and $\mathrm{G}_{k'}$ are symmetric with each other.
- I_{sh}, $I_{\mathrm{sh},k}$, $I_{\mathrm{sh},k'}$—the current that flows on the outer part of the outer conductor of the transmission line T_k. All of $I_{\mathrm{sh},k}$'s equal I_{sh}.
- iC—the inner conductor of a shielded loop, as shown in Fig. 1(a).
- j—the imaginary unit $\sqrt{-1}$.
- L_{eq}—the equivalent inductance of the inductor–capacitor parallel combination at the IMax-resonant frequency ω_{IMax}; not used in this section.
- L_{M}—the inductor to completely suppress the loop current of a shielded loop; in this case the preamplifier A is not connected. Not used in this section.
- L_{te}—the inductance to completely suppress the loop current of a shielded loop derived according to Propositions 1–4; not used in this section.
- L_{sh}—the loop inductance of the surface oSo on a shielded loop, typically given by (1) for a circular loop.
- l_k, l—the length of transmission line T_k; when all transmission lines have the same length, the length is denoted as l.
- M—the matching network between the shielded loop and the amplifier A.
- N—the number of gaps on half the loop.

- O, O'—the two nodes of the antenna output.
- oSi, oSo—the inner and the outer surfaces of the outer conductor of a shielded loop, as shown in Fig. 1(a).
- $\mathbb{R}$—the real number set.
- R_A, R_M, R_O, $R_{o\mathsf{M}}$, R_T—the preamplifier input resistance, the matching network input resistance, the shielded loop resistance, the matching network output resistance, and the resistance presented to the outermost loop, respectively, as shown in Fig. 2(e).
- R_sh—the loop resistance.
- SWR—standing wave ratio.
- T—the circuit formed by transmission lines and tuning components on a shielded loop, as shown in Fig. 2(b), (c), (d).
- T_k—transmission line section as shown in Fig. 2.
- U_0^+, U_0^-—the forward- and backward-propagating wave voltage in a transmission line.
- $U_S(x)$—the voltage at surface S at position x; simplified to $U[S]$ when the specific position is not relevant.
- $U[P]$—the voltage at point or surface P; written as $U_P(x)$ when position x must be specified.
- $U[P,Q]$—the voltage difference between points P and Q.
- V, V_k—the equivalent voltage source of the shielded loop and the equivalent voltage source on transmission line T_k.
- X_A, X_M, X_O, $X_{o\mathsf{M}}$, X_T—the preamplifier input reactance, the matching network input reactance, the shielded loop reactance, the matching network output reactance, the reactance presented to the outmost loop, respectively, as shown in Fig. 2(e).
- $X_{\text{sh},k}$, X_sh—the reactance of (section k of) the loop inductor. $\sum_{k,k'} X_{\text{sh},k} = X_\text{sh}$.
- $X_{\widetilde{\mathsf{T}},11}$, $X_{\widetilde{\mathsf{T}},12}$, $X_{\widetilde{\mathsf{T}},21}$, $X_{\widetilde{\mathsf{T}},22}$—the imaginary parts of the elements of the impedance matrix of the half-circuit $\widetilde{\mathsf{T}}$ as shown in Fig. 2(e).
- X_te——the reactance required to completely suppress the loop current of a shielded loop derived according to Propositions 1–4; not used in this section.
- Y_0, Z_0—the characteristic admittance and impedance of a transmission line, respectively; all transmission lines are assumed to have the same characteristic impedance. $Y_0 Z_0 = 1$.
- Z_A, Z_M, Z_O, $Z_{o\mathsf{M}}$, Z_T—the preamplifier input impedance, the matching network input impedance, the shielded loop impedance, the matching network output impedance, and the impedance presented to the outmost loop, respectively, as shown in Fig. 2(e).
- $Z_{\text{sh},k}$, Z_sh—the loop impedance of each section and the total loop impedance, $Z_\text{sh} = R_\text{sh} + \mathrm{j}X_\text{sh} = R_\text{sh} + \mathrm{j}\omega L_\text{sh}$.
- $\mathbf{Z}_{\widetilde{\mathsf{T}}}$—the impedance matrix of the half-circuit $\widetilde{\mathsf{T}}$, i.e., half of the circuit formed by transmission lines and tuning components on a shielded loop, as shown in Fig. 2(e).
- β, β_IMax—wave propagation phase constants.
- Γ_A, Γ_sh—power wave reflection coefficients defined in (S8) and (S9).
- ω—angular frequency, $\omega = 2\pi f$; in this article referred to as "frequency".
- ω_IMax—the IMax-resonant frequency as defined in Definition 1. $\omega_\text{IMax} = 2\pi f_\text{IMax}$.
- ω_X0—the X0-resonant frequency as defined in Definition 2. $\omega_\text{X0} = 2\pi f_\text{X0}$.

Special notations:

- $\bigcirc^*$—complex conjugation.
- $\widetilde{\bigcirc}$—half quantity; for capacitance $\widetilde{C} = 2C$, voltage $\widetilde{V} = V/2$, impedance $\widetilde{Z} = Z/2$.

2.4 Proof

Since all shielded loops we consider are reflectionally symmetric, the equivalent half circuit can be drawn as Fig. 2(b). The equivalent half circuits of 2- and 3-gap shielded loops can be drawn as Fig. 2(c), (d) following the same procedure.

First, the circuits shown in Fig. 2(a)–(d) can be represented as two-port networks. The loop current on each segment is equal, so that

$$\begin{aligned}&U\left[1_1,1_2\right]+U\left[1_3,1_4\right]+\cdots+U\left[1_{2N-1},1_{2N}\right]\\&\quad=I_{\text{sh}}\left(\widetilde{Z}_{\text{sh},1}+\cdots+\widetilde{Z}_{\text{sh},N}\right)+\widetilde{V}_1+\cdots+\widetilde{V}_N\\&\quad=I_{\text{sh}}\widetilde{Z}_{\text{sh}}+\widetilde{V}\,,\end{aligned}$$

where $U[P,Q]=U[P]-U[Q]$. Therefore all the nodes where I_{sh} flows into and out of T_k, $1\le k\le N$ can be gathered into one port, as shown in Fig. 2(e). This port is designated numbered 1 with its nodes labelled $1_1,\ldots,1_{2N+1}$. The other port is numbered 2, of which the nodes are numbered 2_1 and 2_2. 2_1 coincides with O. 2_2 coincides with 1_{2N}. Following this observation, the transmission lines T_k, all lossless, can be gathered into a network $\widetilde{\mathsf{T}}$, of which the impedance parameters can be written as[1]

$$\mathbf{Z}_{\widetilde{\mathsf{T}}}=\mathrm{j}\begin{bmatrix}X_{\widetilde{\mathsf{T}},11} & X_{\widetilde{\mathsf{T}},12}\\ X_{\widetilde{\mathsf{T}},21} & X_{\widetilde{\mathsf{T}},22}\end{bmatrix}, \tag{S7}$$

where $X_{\widetilde{\mathsf{T}},11}$, $X_{\widetilde{\mathsf{T}},12}$, $X_{\widetilde{\mathsf{T}},21}$ and $X_{\widetilde{\mathsf{T}},22}$ are real numbers. The above can be summarized in Lemma 1.

Lemma 1. *The equivalent circuits of shielded loops can be represented by Fig. 2(e) where the impedance matrix of $\widetilde{\mathsf{T}}$ is specified by* (S7).

The impedance seen by $\widetilde{\mathsf{T}}$ at port 1 is denoted $Z_{\widetilde{\mathsf{T}}}$. It follows naturally that $Z_{\widetilde{\mathsf{T}}}=\widetilde{Z}_{\mathsf{T}}$ as the input impedance of half T at port 1 equals half the input impedance of T at the port where the loop impedance is connected (refer to Fig. 2). Clearly, $\widetilde{Z}_{\mathsf{T}}$ is a function of $\widetilde{Z}_{\mathsf{M}}$, denoted as $\widetilde{Z}_{\mathsf{T}}=\widetilde{Z}_{\mathsf{T}}\left(\widetilde{Z}_{\mathsf{M}}\right)$. $\widetilde{Z}_{\mathsf{T}}(\infty)$ means $\widetilde{Z}_{\mathsf{M}}$ is disconnected from $\widetilde{\mathsf{T}}$.

Then there is Lemma 2. This lemma shows IMax resonance is equivalent to cancelling the reactances of loop inductance and transmission lines, a definition used by Ruytenberg et al.[2,3]

Lemma 2. *$|I_{\text{sh}}|$ reaches its possible minimum or maximum when $\widetilde{X}_{\mathsf{T}}\left(\widetilde{Z}_{\mathsf{M}}\right)+\widetilde{X}_{\text{sh}}=0$, where $\mathrm{Re}\,\widetilde{Z}_{\mathsf{M}}>0$ or $\widetilde{Z}_{\mathsf{M}}=\infty$.*

Proof. When $\widetilde{\mathsf{M}}$ is disconnected from $\widetilde{\mathsf{T}}$, $\widetilde{R}_{\mathsf{T}}=0$. For a certain value of $\widetilde{V}$, the loop current $|I_{\text{sh}}|$ reaches its maximum when $\widetilde{X}_{\mathsf{T}}(\infty)=-\widetilde{X}_{\text{sh}}$.

When $\widetilde{\mathsf{M}}$ is connected to $\widetilde{\mathsf{T}}$, but the amplifier $\widetilde{\mathsf{A}}$ is disconnected from $\widetilde{\mathsf{M}}$, there is still $\widetilde{R}_{\mathsf{T}}=0$. $|I_{\text{sh}}|$ reaches its maximum when $\widetilde{X}_{\mathsf{T}}\left(\widetilde{Z}_{\mathsf{M}}\right)=-\widetilde{X}_{\text{sh}}$.

When $\widetilde{\mathsf{M}}$ is connected to $\widetilde{\mathsf{T}}$ and the amplifier $\widetilde{\mathsf{A}}$ is connected to $\widetilde{\mathsf{M}}$, $\widetilde{R}_{\mathsf{T}}>0$. In this case, $\widetilde{Z}_{\mathsf{T}}$ is constrained by $\widetilde{Z}_{\mathsf{A}}$, $\widetilde{Z}_{o\mathsf{M}}$, and $\widetilde{Z}_{\text{sh}}$. Define power wave reflection coefficients[1,4]

$$\Gamma_{\text{sh}}=\frac{\widetilde{Z}_{\mathsf{T}}-\widetilde{Z}_{\text{sh}}^*}{\widetilde{Z}_{\mathsf{T}}+\widetilde{Z}_{\text{sh}}}, \tag{S8}$$

$$\Gamma_{\mathsf{A}}=\frac{\widetilde{Z}_{\mathrm{oM}}-\widetilde{Z}_{\mathsf{A}}^*}{\widetilde{Z}_{\mathrm{oM}}+\widetilde{Z}_{\mathsf{A}}}. \tag{S9}$$

Because both $\widetilde{\mathsf{T}}$ and $\widetilde{\mathsf{M}}$ are lossless, there is[1,4]:

$$|\Gamma_{\text{sh}}|=|\Gamma_{\mathsf{A}}|\,. \tag{S10}$$

In typical applications, $\widetilde{Z}_{\mathrm{oM}}$ and $\widetilde{Z}_{\mathsf{A}}$ are determined in advance. Thus $|\Gamma_{\text{sh}}|$ and $|\Gamma_{\mathsf{A}}|$ are also determined in advance, and $0\le|\Gamma_{\text{sh}}|=|\Gamma_{\mathsf{A}}|<1$. The relation between I_{sh} and V is

$$I_{\text{sh}}=\frac{\widetilde{V}}{\widetilde{Z}_{\text{sh}}+\widetilde{Z}_{\mathsf{M}}}=\frac{V}{Z_{\text{sh}}+Z_{\mathsf{M}}}=V\cdot\frac{1-\Gamma_{\text{sh}}}{2R_{\text{sh}}}\,. \tag{S11}$$

Constrained by (S10), there is

$$|I_{\rm sh}| \geq |V| \cdot \frac{1-|\Gamma_{\rm sh}|}{2R_{\rm sh}}. \tag{S12}$$

The minimum is reached when $\arg\Gamma_{\rm sh} = 0$. According to (S8), this implies $\widetilde{X}_{\mathsf{T}}\left(\widetilde{Z}_{\mathsf{M}}\right) = -\widetilde{X}_{\rm sh}$. □

The proof of Lemma 3 is provided in Supplementary Information section 2.5 with supporting diagrams in Supplementary Fig. S3.

Lemma 3. *At and near $\omega_{\rm IMax}$, the impedance matrix of $\widetilde{\mathsf{T}}$ exists. Especially, when no tuning components exist,*

$$Z_{\widetilde{\mathsf{T}},11} = \sum_k Z_0 \coth(\mathrm{j}\beta l_k), \tag{S13a}$$

$$Z_{\widetilde{\mathsf{T}},12} = Z_{\widetilde{\mathsf{T}},21} = Z_0/\sinh(\mathrm{j}\beta l_N), \tag{S13b}$$

$$Z_{\widetilde{\mathsf{T}},22} = Z_0 \coth(\mathrm{j}\beta l_N). \tag{S13c}$$

where l_k is the length of T_k, $\mathrm{j}\beta$ is the wave propagation constant in T_k, Z_0 is the characteristic impedance of T_k.

Theorem 4 is central to deriving Propositions 1–4.

Theorem 4. *Let $\widetilde{\mathsf{T}}$ be lossless. For an IMax-resonant shielded loop, at $\omega_{\rm IMax}$:*

- *If $\widetilde{X}_{\mathsf{O}} + \widetilde{X}_{\mathsf{M}} = 0$ where $\widetilde{Z}_{\mathsf{M}} \neq \infty$, then*

$$\widetilde{Z}_{\mathsf{T}}\left(\widetilde{Z}_{\mathsf{M}}\right) = \frac{\widetilde{R}_{\mathsf{O}}\widetilde{R}_{\rm sh}}{\widetilde{R}_{\mathsf{M}}} - \mathrm{j}\widetilde{X}_{\rm sh}. \tag{S14}$$

- *If $\widetilde{X}_{\mathsf{T}}\left(\widetilde{Z}_{\mathsf{M}}\right) + \widetilde{X}_{\rm sh} = 0$ where $\widetilde{Z}_{\mathsf{M}} \neq \infty$, then $\widetilde{X}_{\mathsf{O}} + \widetilde{X}_{\mathsf{M}} = 0$.*

Proof. At resonant frequency $\omega_{\rm IMax}$, $|I_{\rm sh}|$ reaches its maximum. Lemma 2 dictates $\widetilde{X}_{\mathsf{T}}(\infty) + \widetilde{X}_{\rm sh} = 0$.

Lemma 3 states the impedance matrix of $\widetilde{\mathsf{T}}$ exists. Since $\widetilde{\mathsf{T}}$ is lossless, its impedance matrix can be written as (S7). By the definition of an impedance matrix, $X_{\widetilde{\mathsf{T}},11} = \widetilde{X}_{\mathsf{T}}(\infty)$.[1] Since $\widetilde{X}_{\mathsf{T}}(\infty) + \widetilde{X}_{\rm sh} = 0$, $X_{\widetilde{\mathsf{T}},11} = -\widetilde{X}_{\rm sh}$. The shielded loop impedance at port OO' is

$$\widetilde{Z}_{\mathsf{O}} = \mathrm{j}X_{\widetilde{\mathsf{T}},22} + \frac{X_{\widetilde{\mathsf{T}},12}X_{\widetilde{\mathsf{T}},21}}{\mathrm{j}X_{\widetilde{\mathsf{T}},11} + \widetilde{R}_{\rm sh} + \mathrm{j}\widetilde{X}_{\rm sh}}, \tag{S15}$$

which reduces to

$$\widetilde{Z}_{\mathsf{O}} = \mathrm{j}X_{\widetilde{\mathsf{T}},22} + \frac{X_{\widetilde{\mathsf{T}},12}X_{\widetilde{\mathsf{T}},21}}{\widetilde{R}_{\rm sh}}. \tag{S16}$$

Hence $\widetilde{X}_{\mathsf{O}} = X_{\widetilde{\mathsf{T}},22}$ and $\widetilde{R}_{\mathsf{O}}\widetilde{R}_{\rm sh} = X_{\widetilde{\mathsf{T}},12}X_{\widetilde{\mathsf{T}},21} \in \mathbb{R}\setminus\{0\}$. The impedance of $\widetilde{\mathsf{T}}$ presented at port 1 is

$$\widetilde{Z}_{\mathsf{T}} = \mathrm{j}X_{\widetilde{\mathsf{T}},11} + \frac{X_{\widetilde{\mathsf{T}},12}X_{\widetilde{\mathsf{T}},21}}{\mathrm{j}X_{\widetilde{\mathsf{T}},22} + \widetilde{R}_{\mathsf{M}} + \mathrm{j}\widetilde{X}_{\mathsf{M}}}. \tag{S17}$$

Former statement: if $\widetilde{X}_{\mathsf{O}} + \widetilde{X}_{\mathsf{M}} = 0$, $\widetilde{Z}_{\mathsf{T}}$ reduces to

$$\widetilde{Z}_{\mathsf{T}} = \frac{\widetilde{R}_{\mathsf{O}}\widetilde{R}_{\rm sh}}{\widetilde{R}_{\mathsf{M}}} - \mathrm{j}\widetilde{X}_{\rm sh},$$

so the former statement is proven.

Latter statement: to show $\widetilde{X}_{\mathsf{T}}+\widetilde{X}_{\mathrm{sh}}=0 \Rightarrow \widetilde{X}_{\mathsf{O}}+\widetilde{X}_{\mathsf{M}}=0$, apply $\widetilde{X}_{\mathsf{T}}\left(\widetilde{Z}_{\mathsf{M}}\right)+\widetilde{X}_{\mathrm{sh}}=0$ and $X_{\widetilde{\mathsf{T}},11}=-\widetilde{X}_{\mathrm{sh}}$ to (S17). Then

$$\operatorname{Im}\frac{X_{\widetilde{\mathsf{T}},12}X_{\widetilde{\mathsf{T}},21}}{\mathrm{j}X_{\widetilde{\mathsf{T}},22}+\widetilde{R}_{\mathsf{M}}+\mathrm{j}\widetilde{X}_{\mathsf{M}}}=0. \tag{S18}$$

As $X_{\widetilde{\mathsf{T}},12}X_{\widetilde{\mathsf{T}},21}=\widetilde{R}_{\mathsf{O}}\widetilde{R}_{\mathrm{sh}}\in\mathbb{R}\setminus\{0\}$, for (S18) to hold, the only possibility is $\mathrm{j}X_{\widetilde{\mathsf{T}},22}+\mathrm{j}\widetilde{X}_{\mathsf{M}}=0$. Notice (S16) shows $\widetilde{X}_{\mathsf{O}}=X_{\widetilde{\mathsf{T}},22}$. Then $\widetilde{X}_{\mathsf{O}}+\widetilde{X}_{\mathsf{M}}=0$, proving the latter statement. □

From this point onward, the aforementioned propositions follow directly. They are written here in a different order and in forms different from but equivalent to those in the section "Propositions".

Regarding $\widetilde{X}_{\mathsf{O}}=X_{\widetilde{\mathsf{T}},22}$, note that $X_{\widetilde{\mathsf{T}},22}$ is the output reactance $\widetilde{X}_{\mathsf{O}}$ when port 1 is disconnected from all components, i.e., all nodes $1_1, 1_2, \ldots, 1_{2N}$ are disconnected from $\widetilde{Z}_{\mathrm{sh}}$. Tuning components are not involved in the proof of Proposition 4, so $\widetilde{X}_{\mathsf{O}}=X_{\widetilde{\mathsf{T}},22}$ holds whether tuning components exist or not.

Proposition 2. *Whether or not tuning components exist, at ω_{IMax}, $\widetilde{X}_{\mathsf{O}}$ does not change when the loop inductors $\widetilde{L}_{\mathrm{sh}}$ are disconnected from the circuit.*

Theorem 4 states that, for an IMax-resonant shielded loop, at ω_{IMax}, $\widetilde{X}_{\mathsf{O}}+\widetilde{X}_{\mathsf{M}}=0 \Leftrightarrow \widetilde{X}_{\mathsf{T}}\left(\widetilde{Z}_{\mathsf{M}}\right)+\widetilde{X}_{\mathrm{sh}}=0$. Lemma 2 implies that $|I_{\mathrm{sh}}|$ reaches its maximum or minimum if and only if $\widetilde{X}_{\mathsf{O}}+\widetilde{X}_{\mathsf{M}}=0$. According to (S14), $\widetilde{R}_{\mathsf{T}}\propto 1/\widetilde{R}_{\mathsf{M}}$. For $|I_{\mathrm{sh}}|$ to reach its minimum, $\widetilde{R}_{\mathsf{T}}$ must be high, so $\widetilde{R}_{\mathsf{M}}$ must be low. Then there is Proposition 1.

Proposition 1. *For an IMax-resonant shielded loop, at ω_{IMax}, R_{M} must be low and positive, and $X_{\mathsf{M}}+X_{\mathsf{O}}=0$.*

Lemma 3 and Proposition 2 imply that as long as no tuning component is directly connected to T_N or $\mathrm{T}_{N'}$, (S13c) holds. Assumption (iv) requires $\beta_{\mathrm{IMax}}l_N<\pi/2$. This means $X_{\widetilde{\mathsf{T}},22}<0$. Using $\widetilde{X}_{\mathsf{O}}=X_{\widetilde{\mathsf{T}},22}$ leads to Proposition 3.

Proposition 3. *When neither T_N nor $\mathrm{T}_{N'}$ is directly connected to a tuning component, $X_{\mathsf{M}}=-X_{\mathsf{O}}>0$ is necessary to minimize $|I_{\mathrm{sh}}|$.*

In the special case of shielded loops without tuning components, an interesting relation emerges, as stated in Proposition 4.

Proposition 4. *For an IMax-resonant shielded loop without tuning components at ω_{IMax} in which all T_k's have equal lengths $l_1=\cdots=l_N=l$, a low-loss inductor is needed to minimize $|I_{\mathrm{sh}}|$, where the inductance $\widetilde{L}=\widetilde{L}_{\mathrm{sh}}/N$ and $\widetilde{R}<\widetilde{R}_{\mathsf{O}}$.*

Proof. At ω_{IMax}, $\widetilde{X}_{\mathsf{T}}(\infty)+\widetilde{X}_{\mathrm{sh}}=0$, as stated in Lemma 2. Since $\widetilde{X}_{\mathsf{T}}(\infty)=X_{\widetilde{\mathsf{T}},11}$, according to Lemma 3, there is

$$\begin{aligned}-\widetilde{X}_{\mathrm{sh}}=\widetilde{X}_{\mathsf{T}}(\infty)&=\operatorname{Im}\sum_k Z_0\coth\left(\mathrm{j}\beta_{\mathrm{IMax}}l_k\right)\\&=-NZ_0\cot\left(\beta_{\mathrm{IMax}}l\right).\end{aligned} \tag{S19}$$

Assumption (iv) implies the right side of (S19) is negative, so $\widetilde{X}_{\mathrm{sh}}>0$.

Lemma 3 and Theorem 4 imply

$$-\widetilde{X}_{\mathsf{M}}=\widetilde{X}_{\mathsf{O}}=X_{\widetilde{\mathsf{T}},22}=-Z_0\cot\left(\beta_{\mathrm{IMax}}l\right). \tag{S20}$$

Equations (S19) and (S20) lead to $\widetilde{X}_{\mathsf{M}}=\widetilde{X}_{\mathrm{sh}}/N$. Because $\widetilde{X}_{\mathrm{sh}}>0$, $\widetilde{X}_{\mathsf{M}}>0$. Thus $\widetilde{Z}_{\mathsf{M}}$ is inductive and

$$\widetilde{L}_{\mathsf{M}}=\widetilde{L}_{\mathrm{sh}}/N. \tag{S21}$$

$\widetilde{R}<\widetilde{R}_{\mathsf{O}}$ follows from (S14). □

Finally, all derivations presented thus far in the "Derivation" section have used half-circuit analysis. Replacing all half quantities with their full-circuit counterparts yields the corresponding full-circuit propositions in Supplementary Information §2.1.

2.5 Proof of Lemma 3

We mainly consider the case where no tuning component exists, in which the equivalent circuits of an N-gap shielded loop are shown in Fig. S3(a) and Fig. S3(b).

Denote the voltage at node P_1 as $U[P_1]$; $U[P_1,P_2] = U[P_1] - U[P_2]$; the voltage at port 1 as $u_1 = U[1_1, 1_2] + U[1_3, 1_4] + \cdots + U[1_{2N-1}, 1_{2N}]$; the voltage at port 2 as $u_2 = U[2_1, 2_2]$. The impedance matrix of $\widetilde{\mathrm{T}}$ exists if $Z_{\widetilde{\mathrm{T}},11}$, $Z_{\widetilde{\mathrm{T}},22}$, $Z_{\widetilde{\mathrm{T}},21}$, $Z_{\widetilde{\mathrm{T}},22}$ are finite. An open-terminated transmission line shorter than 1/4 wavelength is referred to as an "STL".

Claim. For the structures in Fig. S3(c), (d), although oSo_k and iC_k float, $U[\mathrm{oSo}_k]$ and $U[\mathrm{iC}_k]$ can be chosen as 0.

To see this, insert resistors R_ε of infinitesimal conductance $\varepsilon > 0$ between node iC_k and any non-floating node D_k of finite voltage $U[D_k]$. Assumption (v) dictates that the current flowing into and out of the transmission lines must be equal. Therefore the current branches flowing through the left and right sides of node iC_k are i_1, so the current flowing through R_ε must be zero. Then $U[\mathrm{iC}_k] = U[D_k]$ is finite. Now choose D_k as ground; then $U[\mathrm{iC}_k] = 0$. Similarly $U[\mathrm{oSo}_k] = 0$.

Existence of $Z_{\widetilde{\mathrm{T}},22} = u_2/i_2$ *when* $i_1 = 0$. Disconnect port 1. Immediately $Z_{\widetilde{\mathrm{T}},22} = Z_0 \coth(\mathrm{j}\beta l_N)$. It is finite.

Existence of $Z_{\widetilde{\mathrm{T}},21} = u_2/i_1$ *when* $i_2 = 0$. $U[\mathrm{G}_{N-1}, 1_{2N-1}]$ is the input voltage of an STL created by current source i_1. The voltage along a transmission line is[5]

$$U(x) = U_0^+ \left(\mathrm{e}^{-\mathrm{j}\beta x} + \Gamma \mathrm{e}^{\mathrm{j}\beta x}\right),$$

where $U(x)$ is iC's voltage minus oSo's or oSi's voltage; U_0^+ is the forward-propagating voltage; x is the length offset from a reference point $x = 0$, which in this case is port 2; Γ is the traveling wave reflection coefficient.

Port 2 is open-circuited so $\Gamma = 1$. Thus $U[\mathrm{G}_{N-1}, 1_{2N-1}] = U(l_N) = U_o^+\left(\mathrm{e}^{-\mathrm{j}\beta l_N} + \mathrm{e}^{\mathrm{j}\beta l_N}\right)$. The port voltage at port 2 is $u_2 = U(0) = 2U_o^+$. Invoking $-i_1 = I(l_N) = (U_o^+/Z_0)\cdot\left(\mathrm{e}^{-\mathrm{j}\beta l_N} - \Gamma \mathrm{e}^{\mathrm{j}\beta l_N}\right)$ where $I(l_N)$ is the current flowing into the transmission line on iC at $x = l_N$, we get

$$Z_{\widetilde{\mathrm{T}},21} = (u_2/i_1)|_{i_2=0} = Z_0/\sinh(\mathrm{j}\beta l_N),$$

which is finite when $\beta l_N < \pi/2$.

Existence of $Z_{\widetilde{\mathrm{T}},12} = u_1/i_2$ *when* $i_1 = 0$. No transmission line of $\mathrm{T}_1, \mathrm{T}_2, \ldots, \mathrm{T}_{N-1}$ is excited, so $U[1_1, 1_2] + U[1_3, 1_4] + \cdots + U[1_{2N-3}, 1_{2N-2}] = 0$. For nodes $1_{2N-1}, 1_{2N}$, there is $U[1_{2N-1}, 1_{2N}] = U[1_{2N-1}, \mathrm{iC}_{N-1}]$. Therefore $Z_{\widetilde{\mathrm{T}},12} = u_1/i_2 = Z_0/\sinh(\mathrm{j}\beta l_N)$ is finite.

Existence of $Z_{\widetilde{\mathrm{T}},11} = u_1/i_1$ *when* $i_2 = 0$. All floating nodes oSo_k and iC_k can be set as zero voltage. Therefore:

- On T_N, $U[1_{2N-1}, 1_{2N}] = U[1_{2N-1}, \mathrm{iC}_{N-1}]$ is the input voltage of an STL.
- For $\mathrm{T}_{k+1}, \mathrm{T}_{k+2}$ that constitute the section shown in Fig. S3(a) and Fig. S3(b), $U[1_{2k}, 1_{2k+1}] = U[1_{2k}, \mathrm{iC}_k]$ and $U[1_{2k+2}, 1_{2k+3}] = U[\mathrm{iC}_{k+2}, 1_{2k+3}]$ are the input voltages of the STLs. For odd N, iC_0 is explicitly connected to ground, and the reasoning still applies.
- For even N, 1_1 is explicitly connected to ground, so $U[1_1, 1_2] = U[\mathrm{iC}_1, 1_2]$ is the input voltage of an STL.

Thus $Z_{\widetilde{\mathrm{T}},11}$ is the sum of the impedances of N STLs:[5]

$$Z_{\widetilde{\mathrm{T}},11} = \sum_k Z_0 \coth(\mathrm{j}\beta l_k).$$

When tuning components exist, the finiteness of $Z_{\widetilde{\mathrm{T}},mn}, m,n \in \{1,2\}$ can be shown similarly, as long as no infinite voltage or current is created on any node. □

References

1. Pozar, D. M. Chapter 4: Microwave Network Analysis. In *Microwave Engineering*, chap. 4, 165–227 (John Wiley & Sons, Inc., 2011), 4 edn.
2. Ruytenberg, T., Webb, A. & Zivkovic, I. Shielded-coaxial-cable coils as receive and transceive array elements for 7T human MRI. *Magn. Reson. Med.* **83**, 1135–1146, DOI: 10.1002/mrm.27964 (2020).
3. Nohava, L. *et al.* Flexible Multi-Turn Multi-Gap Coaxial RF Coils: Design Concept and Implementation for Magnetic Resonance Imaging at 3 and 7 Tesla. *IEEE Trans. Med. Imag.* **40**, 1267–1278, DOI: 10.1109/TMI.2021.3051390 (2021).
4. Kurokawa, K. Power Waves and the Scattering Matrix. *IEEE Trans. Microw. Theory Techn.* **13**, 194–202, DOI: 10.1109/TMTT.1965.1125964 (1965).
5. Pozar, D. M. Chapter 2: Transmission Line Theory. In *Microwave Engineering*, chap. 2, 48–90 (John Wiley & Sons, Inc., Singapore, 2011), 4 edn.

Manufacturer	Part no.	Type	Attenuation [dB/m]			
			50 MHz	100 MHz	200 MHz	400 MHz
Belden	8216	RG-174	0.19	0.28	0.41	0.62
Belden	83265	RG-178	0.34	0.46	0.62	0.92
Belden	84316	RG-316	0.18	0.27	0.40	0.57
Huber+Suhner	K_01152-07	—	—	—	1.040	1.501
Huber+Suhner	RG_174_/U	RG-174	—	0.284	0.404	0.575
Huber+Suhner	ENVIROFLEX_178	RG-178	—	0.467	0.674	0.979
Huber+Suhner	ENVIROFLEX_316	RG-316	—	0.247	0.359	0.527

Table S1. Examples of cable loss. Data are from Belden Inc. (St. Louis, MO 63105, USA) and Huber+Suhner AG (9100 Herisau, Appenzell Ausserrhoden, Switzerland).

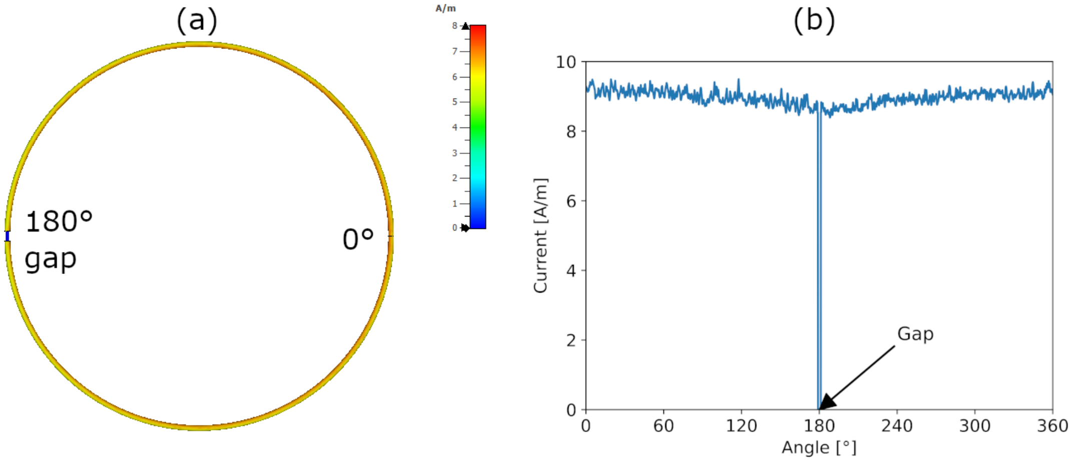

Figure S1. (a) Simulated loop current of a one-gap shielded loop without tuning capacitors. Diameter 16 cm, RG-316, dielectric PTFE, $\varepsilon = 2.1$, $\tan\delta = 2 \times 10^{-4}$; gap 4 mm, loop output gap 10 mm (invisible here). Simulator CST Studio Suite 2025 (Dassault Systèmes; Villacoublay, France), frequency-domain solver, tetrahedral mesh, simulation space 300 mm × 300 mm × 300 mm, open boundary. 528 978 tetrahedra; simulated resonance frequency 65.44 MHz. (b) The current distribution on the loop's outer surface oSo. At this frequency the loop circumference is 0.110× aerial wavelength so the current distributes evenly along the loop.

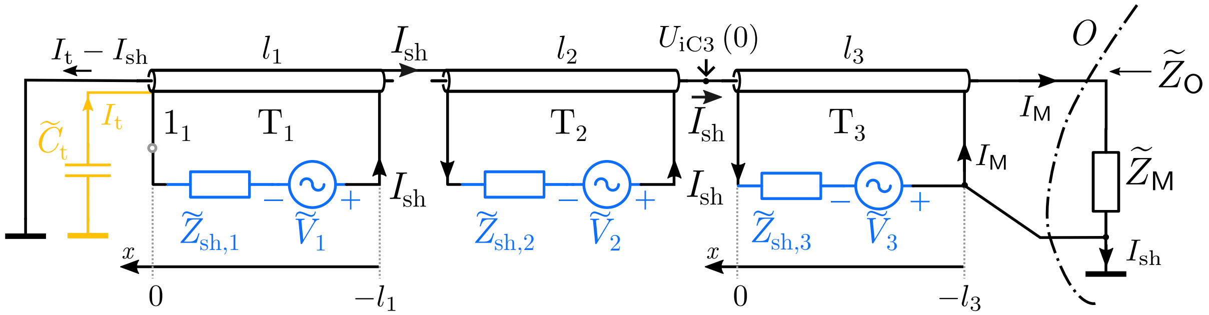

Figure S2. Half of the equivalent circuit of a 3-gap shielded loop, for directly calculating I_{sh} vs. $\widetilde{Z}_M$.

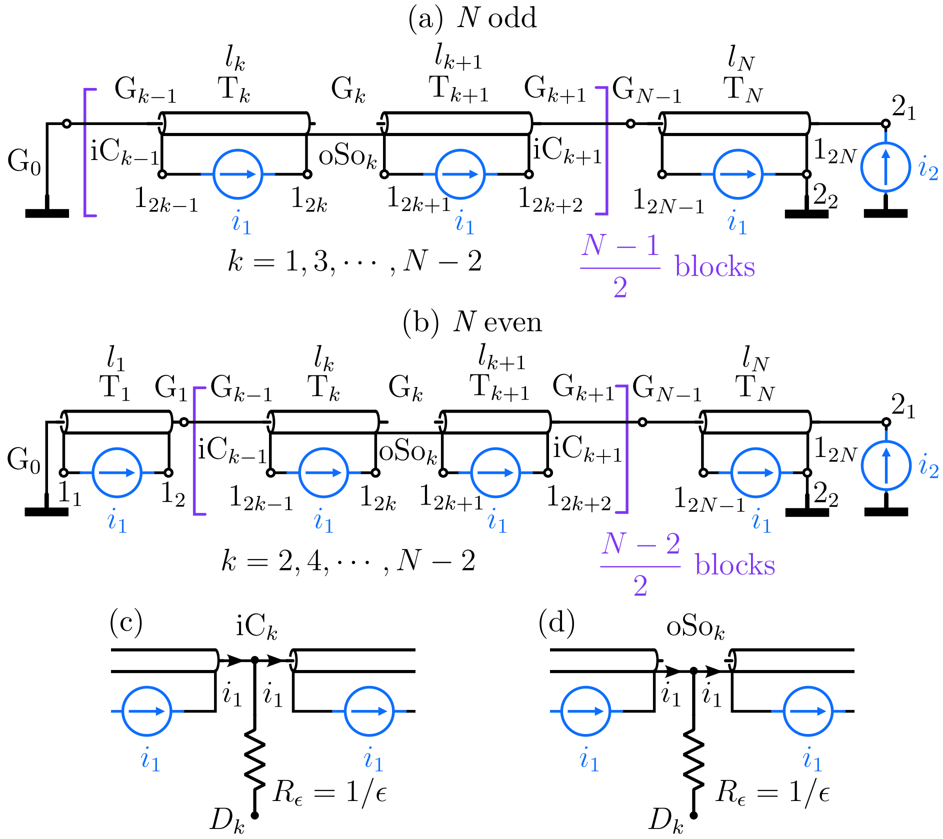

Figure S3. Equivalent circuit of an N-gap shielded loop without tuning components when (a) N is odd and when (b) N is even. $N \geq 1$. 1_{2N} and 2_2 are the same node. Transmission line T_k has length l_k. (c) Establishing $U[\mathrm{iC}_k] = U[D_k]$ by inserting a resistor R_ε of infinitesimal conductance ε between iC_k and any non-floating node D_k. (d) Establishing $U[\mathrm{oSo}_k] = U[D_k]$. D_k in (c), (d) can be chosen as ground.
